# Crystal Engineering and Photomagnetic Studies of CN-Bridged Coordination Polymers Based on Octacyanidometallates(IV) and [Ni(cyclam)]^2+^

**DOI:** 10.1021/acs.inorgchem.2c01629

**Published:** 2022-08-23

**Authors:** Michał Heczko, Ewa Sumińska, Dawid Pinkowicz, Beata Nowicka

**Affiliations:** Faculty of Chemistry, Jagiellonian University, Gronostajowa 2, 30-387 Kraków, Poland

## Abstract

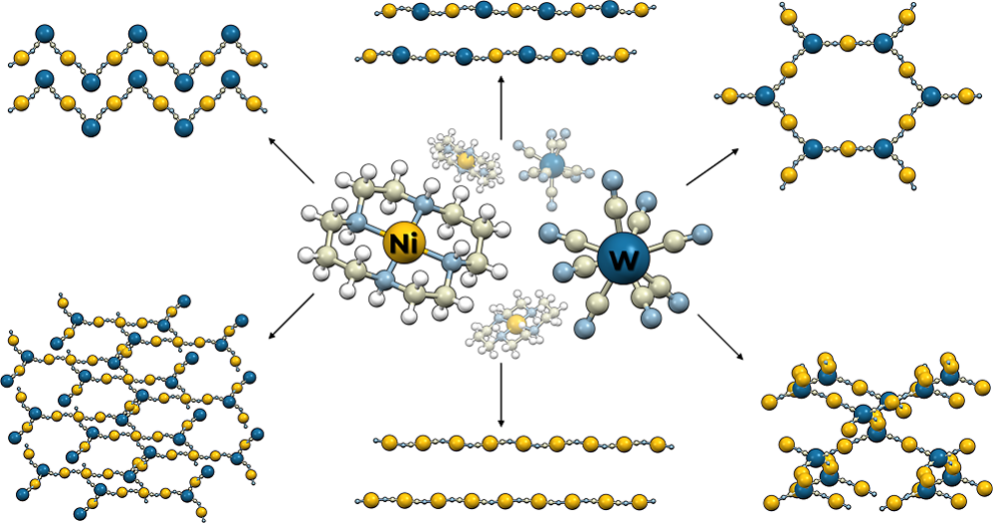

A series of new CN-bridged coordination networks of different dimensionality and topology was obtained through the modification of reaction conditions between [Ni(cyclam)]^2+^ (cyclam = 1,4,8,11-tetraazacyclotetradecane) and [W(CN)_8_]^4–^. The factors determining the reaction pathway are temperature and addition of the LiCl electrolyte. The products include three negatively charged frameworks incorporating Li^+^ guests: the 1D Li_2_[Ni(cyclam)][W(CN)_8_]·6H_2_O (**1**) straight chain, the 1D Li_2_[Ni(cyclam)][W(CN)_8_]·2H_2_O (**2**) zigzag chain, and the 2D Li_2_[Ni(cyclam)]_3_[W(CN)_8_]_2_·24H_2_O (**3**) honeycomb-like network, as well as the 3D two-fold interpenetrating [Ni(cyclam)]_5_[Ni(CN)_4_][W(CN)_8_]_2_·11H_2_O (**4**) network and the 1D [Ni(cyclam)][Ni(CN)_4_]·2H_2_O (**5**) chain, which result from partial decomposition of the starting complexes. Together with the previously characterized 3D [Ni(cyclam)]_2_[W(CN)_8_]·16H_2_O (**6**) network, they constitute the largest family of CN-bridged coordination polymers obtained from the same pair of building blocks. All compounds exhibit paramagnetic behavior because of the separation of paramagnetic nickel(II) centers through the diamagnetic polycyanidometallates. However, the presence of the photomagnetically active octacyanidotungstate(IV) ions allowed observation of the magnetic superexchange after the violet light excitation (405 nm) for compound **3**, which constitutes the first example of the photomagnetic effect in a Ni^II^–[W^IV^(CN)_8_] system. The photomagnetic investigations for fully hydrated and dehydrated sample of **3**, as well as for the isostructural octacyanidomolybdate(IV)-based network are discussed.

## Abstract 2

Six coordination networks of different dimensionality and topology can be obtained from the same pair of building blocks: [Ni(cyclam)]^2+^ and [W(CN)_8_]^4−^ depending on reaction conditions. The negatively charged 2D Li_2_[Ni(cyclam)]_3_[W(CN)_8_]_2_·*n*H_2_O microporous network is the first example of the photomagnetic effect in a Ni^II^−[W^IV^(CN)_8_] system.

## Introduction

Multifunctionality at the molecular level is an appealing pathway to miniaturization. Molecule-based systems comprising organic and inorganic fragments organized into extended structures by chemical bonds and intermolecular interactions may combine a wide range of desired properties including magnetic order,^1^ photosensitivity,^2^ conductivity,^3^ luminescence,^4^ or optical activity.^5^ Cyanido-bridged coordination polymers are an important and versatile group of functional molecule-based materials.^6^ Cyanide ligands allow easy design of hybrid organic–inorganic polynuclear assemblies via the building block approach. Organic ligands may introduce additional properties and can be used to manipulate topology and dimensionality, enabling formation of discrete polynuclear structures, chains, and layers, as well as intricate 3D architectures.^7^ At the same time, cyanide ligands mediate relatively strong interactions between paramagnetic metal centers giving rise to interesting magnetic properties.^8^

Switching of magnetization by visible light is one of the desirable functionalities that can be achieved in molecule-based magnetics. In contrast to classical magnets, molecular systems show great versatility and chemical susceptibility and can be obtained as optically transparent single crystals. The photomagnetic effect is realized by photoexcitation to metastable spin states, which is often connected with modification of existing or appearance of new magnetic exchange pathways. Photomagnetic behavior of octacyanidomolybdate(IV)-based systems was first discovered in a bimetallic Mn^II^–Mo^IV^ chain and interpreted in terms of irreversible Mo^IV^ photo-oxidation.^9^ In a series of Cu^II^–Mo^IV^ assemblies, the mechanism of photo-induced charge transfer was proposed.^10,11^ An alternative concept of Mo^IV^-centered spin transition from singlet to triplet was proposed to explain the photomagnetic behavior of the Zn^II^_2_Mo^IV^ trinuclear molecule.^12^ The excited triplet state trapping by reversible photodissociation of CN was proved to occur in [Mo^IV^(CN)_8_]^4–^ salts.^13^ The octacyanidotungstate(IV) ion was found to show similar photo-induced spin transition to its Mo^IV^ congener in the hexanuclear Mn^II^–M^IV^ and Fe^II^–M^IV^ (M = Mo, W) clusters.^14,15^ In the latter compounds, site-selective photoswitching on both Fe^II^ and M^IV^ metal centers could be achieved by using irradiation with different wavelengths.^15^ Photo-induced ferrimagnetic order with *T*_c_ of 93 K was found in the 3D {[Mn^II^(Him)]_2_[W^IV^(CN)_8_]} framework.^16^ Interestingly, the photomagnetic effect in this network can be deactivated by the absorption of moisture. However, the [W^IV^(CN)_8_]^4–^-based systems are still sparsely investigated in terms of the photomagnetic effect in comparison to those of [Mo^IV^(CN)_8_]^4–^.^1^ Moreover, Ni^II^–[W^IV^(CN)_8_] photomagnetic systems were not reported previously.

The construction of molecule-based materials is usually carried out using the building block self-assembly method. By taking into account the building block characteristics, in particular the geometry, charge, coordination environment of metal centers, and potential ability to create new coordination connections, certain predictions regarding the product dimensionality and topology can be made.^17,18^ In addition to strong coordination bonds, the presence of weaker intermolecular interactions including hydrogen bonds and/or pi-stacking, greatly affects the resulting product structure.^19−22^ Selection of an appropriate solvent as well as time and temperature of the reaction is not less important.^23−25^ Also, the presence and concentration of an additional electrolyte may strongly influence the course of the crystallization process, by increasing the ionic strength of the solution. It often results in the increase of the building blocks’ solubility and the slower crystallization process, which can lead to the formation of larger and better quality product crystals. Moreover, the presence of guest molecules and ions in the solution often results in their incorporation into the product structure, particularly in the case of microporous systems, which can result in the change of network dimensionality or topology.

In our previous studies, we have presented the versatility of the [Ni(cyclam)]^2+/3+^ (cyclam = 1,4,8,11-tetraazacyclotetradecane) cationic building block using it as a linear linker in combination with various polycyanidometallates. It afforded numerous coordination networks differing in dimensionality and topology with a wide range of desirable properties, including long-range magnetic order, microporosity, sorption ability, solvatomagnetic effect,^26−28^ ionic conductivity,^29,30^ and presence of multi-switchable electron-transfer phase transitions with a memory effect.^31^ We have also shown the possibilities of manipulation of topology by incorporation of guest ions or changing reaction conditions.^32,33^ In this work, we present an unprecedented success of crystal engineering: six different networks of varied topologies obtained from [Ni(cyclam)]^2+^ and [W(CN)_8_]^4–^ building blocks with addition of LiCl by varying reactants’ concentrations and reaction temperature. Moreover, we present the first investigation of the photomagnetic effect for a Ni^II^–W^IV^ network, as well as its Ni^II^–Mo^IV^ analogue.

## Experimental Section

### Materials

The K_4_[W(CN)_8_]·2H_2_O precursor complex was synthesized according to the literature procedure.^34^ [Ni(cyclam)(NO_3_)_2_] was prepared according to the modified published method.^35^ Ni(NO_3_)_2_·6H_2_O (535.0 mg, 1.84 mmol) was dissolved in boiling absolute ethanol (10 mL). To the above solution, solid cyclam (368.6 mg, 1.84 mmol) was added which resulted in the immediately precipitating violet product. The suspension was stirred for 10 min and left in the freezer for 1 h (−18 °C). The product was filtered and washed with a small amount of cold acetone (4 °C). Yield: 701.0 mg (99.4%). All other reagents and solvents were commercially available and used as supplied.

### Synthesis of Compounds

#### Li_2_[Ni(cyclam)][W(CN)_8_]·6H_2_O (**1**)

A water solution (5 mL) of [Ni(cyclam)(NO_3_)_2_] (19.2 mg, 0.05 mmol) and LiCl (2.5 g, 58.97 mmol), heated to 46 °C, was added to a water solution (5 mL) containing K_4_[W(CN)_8_]·2H_2_O (29.2 mg, 0.05 mmol) and LiCl (2.5 g, 58.97 mmol) heated to 46 °C. The yellow solution was left for crystallization at room temperature. After 24 h, the yellow plate-shaped crystals of **1** were formed. In order to perform the elemental analysis and IR spectra, the product was filtered and washed with small amount of LiCl water solution (5 mL, 2.5 g) because the compound is unstable in pure water. EA: found: C, 26.23; N, 20.04; H, 4.65, calculated for: C_18_H_38_ClLi_3_N_12_Ni_1_O_7_W_1_ (Li_2_[Ni(cyclam)][W(CN)_8_]·6H_2_O + LiCl·H_2_O): C, 25.94; N, 20.17; H, 4.60. IR ν(CN): 2114.7, 2128.2 cm^–1^. The purity of **1** was additionally confirmed by powder X-ray diffraction measurement for the crystalline sample in LiCl water solution (5 mL, 2.5 g).

#### Li_2_[Ni(cyclam)][W(CN)_8_]·2H_2_O (**2**)

A water solution (5 mL) of [Ni(cyclam)(NO_3_)_2_] (19.2 mg, 0.05 mmol) and LiCl (2.5 g, 58.97 mmol), heated to 105 °C, was added to a water solution (5 mL) of K_4_[W(CN)_8_]·2H_2_O (29.2 mg, 0.05 mmol) and LiCl (2.5 g, 58.97 mmol) heated to 105 °C. The resulting yellow solution was slowly cooled to 40 °C and left for crystallization in a water bath (40 °C). After 24 h, the yellow needle-shaped crystals of **2** were formed. In order to perform the elemental analysis and IR spectra, the product was filtered and washed with small amount of LiCl water solution (5 mL, 2.5 g), due to decomposition that takes place upon even short contact with pure water. EA: found: C, 24.56; N, 19.03; H, 4.82, calculated for: C_18_H_38_Cl_2_Li_4_N_12_Ni_1_O_7_W_1_ (Li_2_[Ni(cyclam)][W(CN)_8_]·2H_2_O + 2LiCl·5H_2_O)): C, 24.69; N, 19.19; H, 4.37. IR ν(CN): 2114.7, 2127.2, 2136.8 cm^–1^. The purity of **2** was additionally confirmed by powder X-ray diffraction measurement for the crystalline sample in LiCl water solution (5 mL, 2.5 g).

#### Li_2_[Ni(cyclam)]_3_[W(CN)_8_]_2_·24H_2_O (**3**)

A water solution (10 mL) of [Ni(cyclam)(NO_3_)_2_] (9.6 mg, 0.025 mmol) and LiCl (1 g, 23.59 mmol) was added at room temperature to a water solution (10 mL) of K_4_[W(CN)_8_]·2H_2_O (9.9 mg, 0.017 mmol) and LiCl (1 g, 23.59 mmol). The resulting suspension was left at room temperature and after 3 days yellow block-shaped crystals of **3** were formed. In order to perform the elemental analysis and IR spectra, the product was filtered, washed with small amount of cold water, and dried in air, which resulted in partial dehydration. EA: found: C, 27.78; N, 19.60; H, 5.82, calculated for: C_46_H_118_Li_2_N_28_Ni_3_O_23_W_2_ (Li_2_[Ni(cyclam)]_3_[W(CN)_8_]_2_·23H_2_O): C, 27.77; N, 19.72; H, 5.98. IR ν(CN): 2103.5, 2123.7 cm^–1^. The purity of **3** was additionally confirmed by powder X-ray diffraction measurement for the crystalline sample suspended in the LiCl solution of the same concentration as the mother liquor.

#### [Ni(cyclam)]_5_[Ni(CN)_4_][W(CN)_8_]_2_·11H_2_O (**4**)

A water solution (10 mL) of [Ni(cyclam)(NO_3_)_2_] (40.0 mg, 0.11 mmol) and LiCl (1 g, 23.59 mmol), heated to 84 °C, was added to a water solution (10 mL) of K_4_[W(CN)_8_]·2H_2_O (40.0 mg, 0.07 mmol) and LiCl (1 g, 23.59 mmol) heated to 84 °C. The resulting yellow solution was slowly cooled to 62 °C and left for crystallization in a water bath. After 2 days, the yellow block-shaped crystals of **4** were formed. In order to perform the elemental analysis and IR spectra, the product was filtered, washed with water, and dried in air, which resulted in partial dehydration. EA: found: C, 34.89; N, 23.16; H, 5.77, calculated for: C_70_H_138_N_40_Ni_6_O_9_W_2_ ([Ni(cyclam)]_5_[Ni(CN)_4_][W(CN)_8_]_2_·9H_2_O): C, 34.97; N, 23.31; H, 5.79. IR ν(CN): 2091.5, 2105.0, 2119.5, 2138.8 cm^–1^. The purity of **4** was additionally confirmed by powder X-ray diffraction measurement for the crystalline sample in water.

#### [Ni(cyclam)][Ni(CN)_4_]·2H_2_O (**5**)

The light pink block-shaped crystals of **5** suitable for single crystal X-ray diffraction were obtained from the filtrate of compound **4** after about 2 weeks at room temperature. Compound **5** was also obtained in an alternative way: a water solution (35 mL) of [Ni(cyclam)(NO_3_)_2_] (40.0 mg, 0.11 mmol) and KNO_3_ (5 g, 49.45 mmol), heated to 65 °C, was added to a water solution (35 mL) of K_2_[Ni(CN)_4_] (26.51 mg, 0.11 mmol) and KNO_3_ (5 g, 49.45 mmol) heated to 65 °C. The yellow solution was slowly cooled to 50 °C and after about 2 h the crystals of **5** were formed. The reaction solution was cooled to the room temperature. In order to perform the elemental analysis and IR spectra, the product was filtered, washed with water, and dried in air, which resulted in partial dehydration. EA: found: C, 39.64; N, 26.06; H, 5.71, calculated for: C_14_H_25_N_8_Ni_2_O_0.5_ ([Ni(cyclam)][Ni(CN)_4_]·0.5H_2_O): C, 39.03; N, 26.01; H, 5.85. IR ν(CN): 2116.6, 2128.2, 2140.7, 2159.0 cm^–1^. The purity of **5** was additionally confirmed by powder X-ray diffraction measurement for crystalline sample in water.

#### [Ni(cyclam)]_2_[W(CN)_8_]·16H_2_O (**6**)

[Ni(cyclam)]_2_[W(CN)_8_]·16H_2_O (**6**) was synthesized according to the published method.^27^

#### Li_2_[Ni(cyclam)]_3_[Mo(CN)_8_]_2_·24H_2_O (**7**)

Li_2_[Ni(cyclam)]_3_[Mo(CN)_8_]_2_·24H_2_O (**7**) was obtained according to the published method.^33^

### Structure Determination

The single-crystal X-ray diffraction measurements for samples **1–5** were performed on a Bruker D8 Quest Eco diffractometer equipped with a Mo Kα radiation source (λ = 0.71073 Å). The structures were solved by direct methods with SHELXT and the refinement was performed using SHELXL.^36^ All non-hydrogen atoms were refined anisotropically. The C–H and N–H hydrogen atoms were placed in idealized positions and refined isotropically using the riding model with *U*_iso_(H) equal to 1.2*U*_eq_ for the C- or N-atoms. H-atoms for heavily disordered water molecules in the structures of **3** and **4** were not considered but included in the empirical formula. In the structures of **1**, **2**, and **5**, the H-atoms of water molecules were found from the electron density map and refined isotropically without restraints. The Li^+^ cations in the structures of **1**, **2,** and **3** were found from the electron density map. The presence of tetrahedral coordination environment was considered in the assignment of Li. Due to high absorption, which effect is not compensated by empirical multi-scan correction, some residual electron density peaks appear around W in the structure of **3**. Graphical representations of the structures were prepared with Mercury CSD 4.3.1 software.^37^ The analysis of coordination polyhedra was performed using SHAPE 2.1.^38^ The crystallographic data are presented in Table 1.

**Table 1:** Selected Crystallographic Data for Compounds **1–5**

compound	**1**	**2**	**3**	**4**	**5**
empirical formula	C_18_H_36_Li_2_N_12_NiO_6_W	C_18_H_28_Li_2_N_12_NiO_2_W	C_46_H_120_Li_2_N_28_Ni_3_O_24_W_2_	C_70_H_142_N_40_Ni_6_O_11_W_2_	C_14_H_28_N_8_Ni_2_O_2_
FW	773.03	700.96	2007.40	2440.19	457.86
T (K)	120	120	100	100	100
crystal system	monoclinic	monoclinic	monoclinic	monoclinic	triclinic
space group	*P*2/*n*	*C*2/*c*	*C*2/*m*	*P*2_1_/*n*	*P*1̅
a (Å)	10.3259(2)	12.2145(10)	26.2103(14)	14.3208(6)	7.7147(7)
b (Å)	9.5362(2)	14.1512(11)	15.3800(8)	14.8322(6)	8.8748(8)
c (Å)	15.5361(3)	14.5322(12)	10.1406(6)	23.1077(9)	15.7304(14)
α (deg)	90	90	90	90	88.656(5)
β (deg)	104.698(1)	94.454(3)	95.268(2)	93.578(2)	85.592(5)
γ (deg)	90	90	90	90	74.727(5)
V (Å^3^)	1479.78(5)	2504.3(4)	4070.6(4)	4898.7(3)	1035.89(16)
*Z*	2	4	2	2	2
ρ_calc_ (mg/m^3^)	1.735	1.859	1.638	1.654	1.468
μ (mm^–1^)	4.573	5.383	3.579	3.538	1.842
*F*(000)	768	1376	2044	2492	480
crystal size (mm)	0.30 × 0.11 × 0.07	0.50 × 0.05 × 0.05	0.20 × 0.12 × 0.06	0.26 × 0.21 × 0.04	0.14 × 0.13 × 0.09
θ range (deg)	3.45–30.6	3.35–28.7	2.43–25.1	2.59–30.5	2.38–27.9
reflections collected	23852	25358	16913	63270	20863
independent	4293	3229	3729	14257	4939
observed	3978	3096	3422	12089	3216
parameters	183	171	259	589	253
*R*_int_	0.037	0.038	0.055	0.041	0.053
GOF on *F*^2^	1.077	1.065	1.059	1.057	1.148
*R*_1_ [*I* > 2σ(*I*)]	0.0254	0.0162	0.0481	0.0386	0.0446
w*R*_2_ (all data)	0.0506	0.0384	0.1284	0.0821	0.0829

### Physical Measurements

Powder X-ray diffraction data were collected on a PANalytical X’Pert PRO MPD diffractometer (samples **1–3** immersed in LiCl water solution of the same concentration as used in syntheses) and on a Bruker D8 Advance Eco diffractometer (samples immersed in pure water, stable in air, and dehydrated), both equipped with a Cu Kα radiation source (λ = 1.541874 Å), in the Debye–Scherrer geometry. The microcrystalline samples were sealed in glass capillaries and measured in the 5–50° 2θ range at room temperature. The reference powder patterns from SC-XRD measurements were generated using Mercury CSD 4.3.1 software.^37^ Elemental analyses of CHN were performed on an ELEMENTAR Vario Micro Cube CHNS analyzer. IR spectra in the range of 4000–500 cm^–1^ were collected on a Thermo Scientific Nicolet iN10 MX FTIR microscope operating in the transmission mode. Thermogravimetric analysis (TGA) was performed on a Mettler Toledo TGA/SDTA 851e analyzer under an argon atmosphere in a temperature range of 35–300 °C with a heating rate of 2 °C·min^–1^. The water sorption/desorption processes were characterized by the dynamic vapor sorption method using the SMS DVS Resolution apparatus. The isotherms for **3** and **4** were measured in a 0–94% relative humidity range at a temperature of 25 °C. Every measurement step was performed until a stable mass was achieved (d*m*/d*t* = 0.002 mg·min^–1^). Magnetic susceptibility measurements were performed on a Quantum Design MPMS-3 Evercool magnetometer in magnetic fields up to 70 kOe. The experimental data were corrected for diamagnetism of the sample and the sample holder. The fully hydrated microcrystalline samples were sealed in double polyethylene bags under pure water (**4**, **5**) or lithium chloride water solution of the same concentration as used in syntheses (**1**, **2**, **3**, **7**) to prevent dehydration. The dehydrated under dry nitrogen flow samples of **3**_**deh**_ and **7**_**deh**_ were protected with paraffin oil and placed in polycarbonate capsules under an argon atmosphere in a glovebox. Samples for photomagnetic measurements were ground to a fine powder and spread onto a colorless adhesive tape in form of a thin layer protected with a Scotch tape. The hydrated phases were additionally protected with small amount of lithium chloride solution and dehydrated forms were treated similarly to bulk samples. In order to prevent **3** and **7** from dehydration in the magnetometer sample chamber, the samples were inserted at 250 K and then vacuum-pumped. The irradiation was performed using 405 and 450 nm laser diodes (both wavelengths fall within the d–d absorption bands of [M(CN)_8_]^4–^ (M = Mo, W) complexes).

## Results and Discussion

### Crystal Engineering

**Figure 1:**
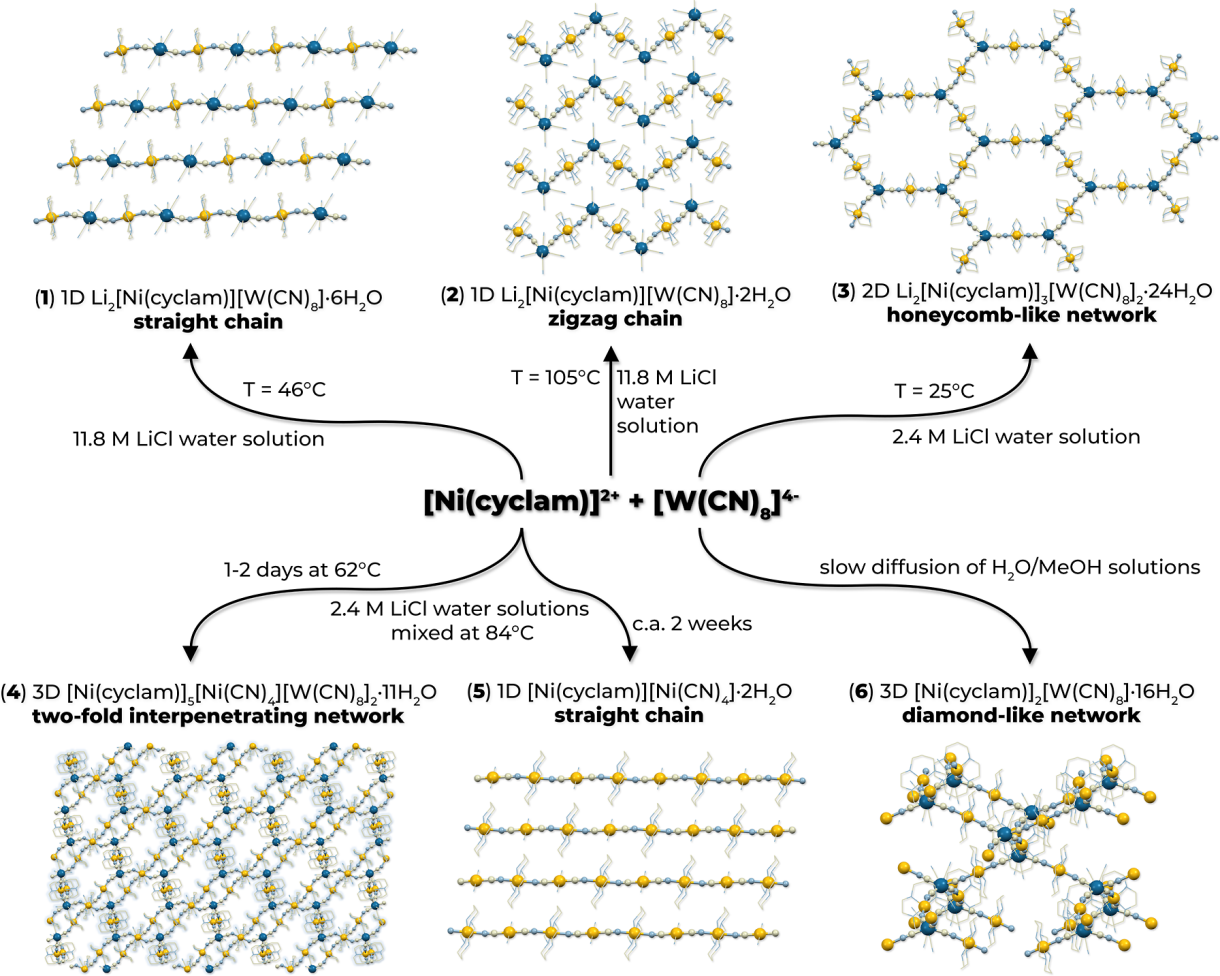
Scheme of reaction pathways between [Ni(cyclam)]^2+^ and [W(CN)_8_]^4–^ building blocks leading to different topology networks; Ni: yellow balls, W: dark blue balls, bridging CN: gray and light blue balls, terminal ligands: sticks. Guest molecules and ions omitted for clarity.

The reaction between [Ni(cyclam)]^2+^ and [W(CN)_8_]^4–^ in water–methanol solution under slow diffusion conditions was found to lead to a 3D diamond-like [Ni(cyclam)]_2_[W(CN)_8_]·16H_2_O network.^27^ The fact that a quick precipitation reaction from the same solution led to a product with a different PXRD pattern was a clear indication of the existence of other possible reaction pathways. In order to investigate that possibility, we performed the reaction between the same building blocks in water solution with the addition of LiCl at various temperatures. All syntheses were preliminarily carried out at the 3:2 ratio between [Ni(cyclam)]^2+^ and [W(CN)_8_]^4–^. The addition of the LiCl electrolyte and elevated reaction temperature were expected to prevent instantaneous precipitation of the product and facilitate crystallization of the thermodynamically most stable network under given reaction conditions. As a result of applied modifications, five new compounds differing in network dimensionality and topology were obtained: a 1D Li_2_[Ni(cyclam)][W(CN)_8_]·6H_2_O (**1**) straight chain and a Li_2_[Ni(cyclam)][W(CN)_8_]·2H_2_O (**2**) zigzag chain, a 2D Li_2_[Ni(cyclam)]_3_[W(CN)_8_]_2_·24H_2_O (**3**) honeycomb-like network, a 3D two-fold interpenetrating [Ni(cyclam)]_5_[Ni(CN)_4_][W(CN)_8_]_2_·11H_2_O (**4**) network, and a 1D [Ni(cyclam)][Ni(CN)_4_]·2H_2_O (**5**) chain. In order to obtain pure products with good reproducibility, the synthetic conditions were optimized in terms of lithium chloride concentration, reaction temperature, and the building blocks’ ratio. The different reaction pathways are summarized in Figure 1.

In the case of compounds **1–3**, the formation of a negatively charged coordination skeleton and incorporation of the Li^+^ cations into the structure is observed. The reaction carried out in highly concentrated LiCl solution (close to saturation at 20 °C) leads to the formation of 1D alternating bimetallic Ni^II^-W^IV^ chains. Depending on the reaction temperature straight (**1**) or zigzag shape (**2**) chain structures are formed. The optimized building block ratio for the synthesis of **1** and **2** is 1:1. The compounds are stable in the mother liquor or concentrated LiCl solution. However, they recrystallize immediately in contact with water, to form a neutral [Ni(cyclam)]_2_[W(CN)_8_]·*n*H_2_O network,^27^ with some unidentified additional phase in the case of **2**, as shown by PXRD (Figures S1 and S2). Such high sensitivity to water is caused by the presence of the guest Li^+^ cations in the structure, which are easily removed by water, causing reorganization of the coordination network. We have observed similar behavior in related compounds containing guest cations.^29,32,33^**1** and **2** remain stable in air when filtered off, which was checked by repeated SC-XRD unit cell determination on dry crystals. Because they cannot be washed with water, dry compounds **1** and **2** contain small amount of hydrated LiCl on the surface. The elemental analysis results show contamination with lithium chloride at ca. 7% for **1** and 20% for **2**. The difference may be caused by the smaller size of crystals of **2**.

The 2D Li_2_[Ni(cyclam)]_3_[W(CN)_8_]_2_·24H_2_O (**3**) honeycomb-like network was obtained at room temperature with lower concentration of LiCl than in the case of 1D compounds **1** and **2**. The stoichiometric 3:2 ratio between the building blocks was found optimal. The reaction initially resulted in fine yellow suspension, which after a few days recrystallized to yellow block-shaped crystals of **3**. Crystals of **3** are stable in the mother liquor or LiCl solution of the same concentration (Figure S3), but disintegrate when placed in water. However, in contrast to compounds **1** and **2**, sample of **3** remains crystalline when filtered off and washed with small amount of water. The elemental analysis has shown that drying in air resulted in the loss of one crystallization water molecule per formula unit. Further dehydration under dry nitrogen flow results in structural transformation, typical for related 2D networks,^26,28^ which is confirmed by changes in the PXRD pattern. The stability of **3** is similar to that of the analogous compound based on the [Mo(CN)_8_]^4–^ building block,^33^ as well as the family of its isotypic M_*x*_[Ni(cyclam)]_3_[Nb(CN)_8_]_2_·*n*H_2_O (M = NH_4_^+^, Li^+^, Na^+^, Mg^2+^, Ca^2+^, Sr^2+^, Ba^2+^, *x* = 1 or 2) congeners.^29,32^

The 3D two-fold interpenetrating [Ni(cyclam)]_5_[Ni(CN)_4_][W(CN)_8_]_2_·11H_2_O (**4**) network was obtained at the same LiCl concentration as compound **3**, but the solutions were mixed at 84 °C and no initial precipitate was observed. The optimal building block ratio for the synthesis of **4** was found to be 1:1. Formation of **4** is the result of partial decomposition of the building blocks and the formation of the [Ni(CN)_4_]^2–^ complex. In contrast to compounds **1–3**, the lithium cations are not embedded in the structure of **4** and therefore its crystals are stable in water as shown by PXRD measurements (Figure S4). The crystals are stable when dried in air, but in the flow of dry nitrogen they disintegrate and small shifts of the PXRD peaks are visible, indicating that some structural changes take place upon dehydration.

If the crystals of **4** are filtered off and the filtrate is left at room temperature, an additional product of the formula [Ni(cyclam)][Ni(CN)_4_]·2H_2_O (**5**) is obtained after about 2 weeks in the form of light pink crystals. The same compound was obtained by a different method and structurally characterized earlier.^39^ We also obtained **5** in an alternative synthesis by mixing equimolar amounts of [Ni(cyclam)]^2+^ and [Ni(CN)_4_]^2–^ dissolved in 1.4 mol·dm^–3^ potassium nitrate water solution at 65 °C. The purity of **5** and its stability in water was checked by PXRD measurements (Figure S5). Upon drying in air, compound **5** losses most of the crystallization water and undergoes structural transformation, which was confirmed by PXRD and EA.

### Structure Description

#### 1D Straight Chain of Li_2_[Ni(cyclam)][W(CN)_8_]·6H_2_O (**1**)

**Figure 2:**
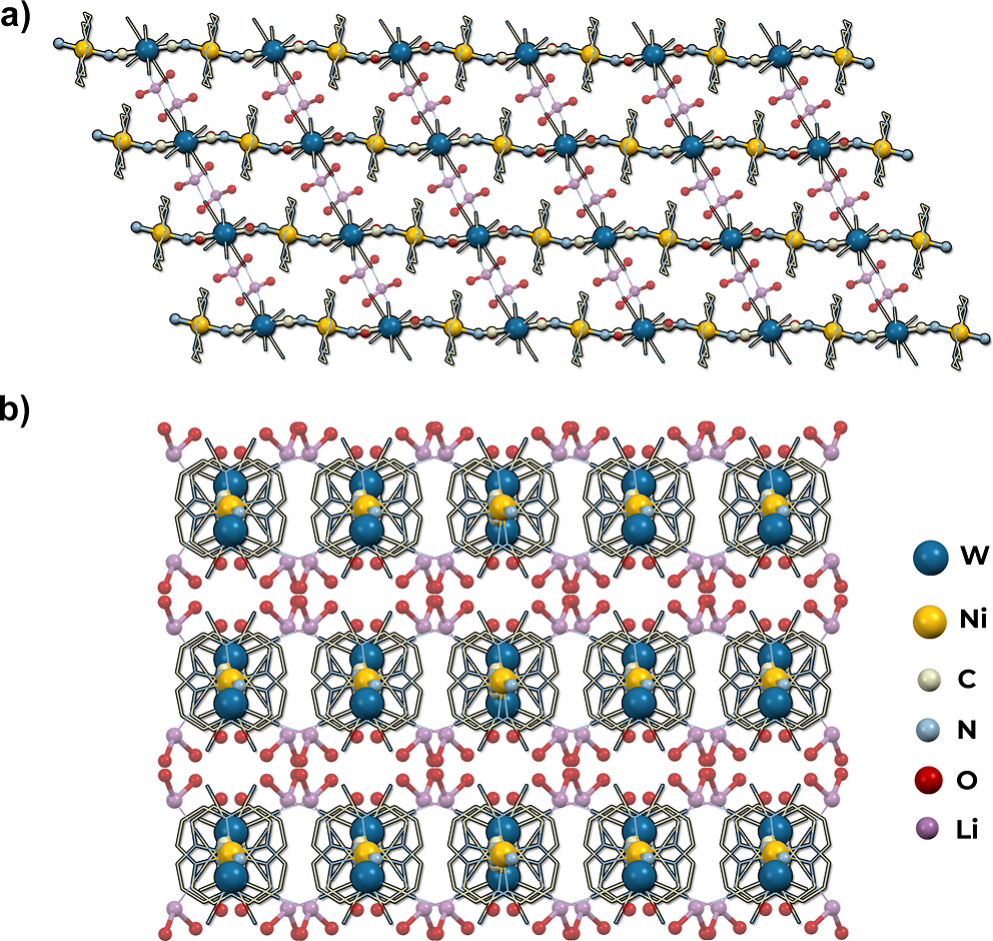
Structure of **1**. View along the [010] direction (a) and along the coordination chains in the [−101] crystallographic direction (b).

Li_2_[Ni(cyclam)][W(CN)_8_]·6H_2_O (**1**) crystallizes in the monoclinic system, the space group *P*2/*n* (Table 1). The asymmetric unit contains 1/2 of the [W(CN)_8_]^4–^ ion located at the two-fold rotation axis, 1/2 of the [Ni(cyclam)]^2+^ ion at the center of inversion, as well as one Li^+^ cation, and three crystallization water molecules in general positions (Figure S6). The W^IV^ ions are coordinated by eight cyanide ligands, two of which are connected to nickel centers and four to lithium cations. The geometry of the [W(CN)_8_]^4–^ building block is close to an ideal square antiprism (*D*_4d_, CShM = 0.166, Table S1). The Ni^II^ ions, equatorially coordinated by four N-atoms of the cyclam ligand and axially by two N-atoms of CN bridges, show slightly distorted octahedral geometries (*O*_h_, CShM = 0.172, Table S2), due to the elongation of the axial bonds and angular distortions caused by coordination of cyclam (Table S4). The lithium cations are surrounded by two water molecules and two cyanido ligands of neighboring octacyanidotungstate(IV) anions, in the geometry close to an ideal tetrahedron (*T*_d_, CShM = 0.780, Table S3). The structure of **1** consists of one-dimensional chains of alternating cyanido-bridged [Ni(cyclam)]^2+^ and [W(CN)_8_]^4–^ moieties that run along the [−101] direction (Figure 2).

The coordination chains are slightly undulating with the Ni1–W1–Ni1 angle equal to 153.17°. The CN bridges are bent (160.0°) resulting in a distance between the Ni and W metal centers of 5.3258 Å (Table S4). The negatively charged chains are linked through lithium ions into layers located on the (020) crystallographic plane. Within the layer, the neighboring chains are separated by 7.49 Å and their relative shift is 7.11 Å along the [−101] direction, whereas the distance between supramolecular layers is equal to 9.54 Å. The presence of semi-coordination bonds between lithium cations and N-atoms of [W(CN)_8_]^4–^ ions of neighboring chains has an impact on the distorted tetrahedral geometry of lithium centers. The distortion is also caused by the presence of hydrogen bonds between the water molecules connected to lithium centers of different layers that results in an O1–Li1–O2 angle equal to 99.39°, which is almost 10° less than for the ideal tetrahedron.

#### 1D Zigzag Chain of Li_2_[Ni(cyclam)][W(CN)_8_]·2H_2_O (**2**)

**Figure 3:**
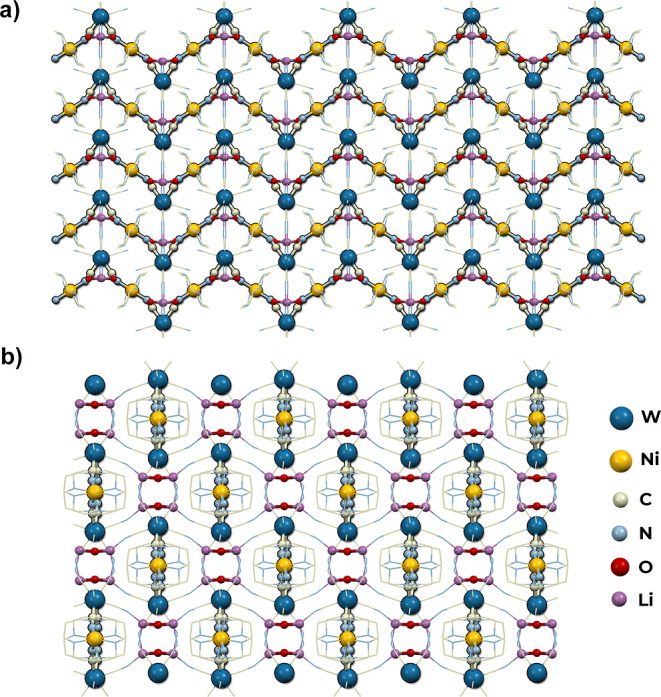
Structure of **2.** View along the [100] direction showing the zigzag shape of the coordination chains (a) and view along the chains in the [001] direction (b).

Li_2_[Ni(cyclam)][W(CN)_8_]·2H_2_O (**2**) crystallizes in the monoclinic system, the space group *C*2/*c*. The asymmetric unit shown in Figure S7 is similar to compound **1**. It is also composed of one-half of the [Ni(cyclam)]^2+^ complex in a special position at the center of inversion, one-half of the [W(CN)_8_]^4–^ ion located at the two-fold axis, and one lithium cation in a general position. However, in contrast to **1** the asymmetric unit of **2** contains not three but only one crystallization water molecule in a general position. As in the structure of **1**, the tungsten center is coordinated by eight cyanide ligands, two of which are connected to nickel ions, but instead of four, only two of the remaining ones are connected to lithium ions. The Ni1 and Li1 coordination environment is the same as in compound **1**. CShM analysis (Tables S2 and S3) shows that for Ni1 octahedral geometry distortion is slightly higher than for **1** (*O*_h_, CShM = 0.195). The geometry of the tungsten complex is different than in **1**, instead of square antiprism (*D*_4d_, CShM = 1.820), the complex is closer to the triangular dodecahedron (*D*_2d_, CShM = 0.319). The structure of **2** also consists of one-dimensional chains that are formed of alternating [Ni(cyclam)]^2+^ and [W(CN)_8_]^4–^ building blocks, but with the distinct zigzag shape (Figure 3) with the Ni1–W1–Ni1 angle of 87.52°. The CN bridges connected to nickel centers are also more bent (155.47°), which result in closer than in **1** distance between neighboring nickel and tungsten metal centers (5.2527 Å). The coordination chains run along the crystallographic *c* direction. Two lithium cations are connected by two water molecules forming [Li_2_(H_2_O)_2_]^2+^ units, which link the chains by semi-coordination bonds to terminal cyanido ligands, resulting in a three-dimensional supramolecular network.

#### 2D Honeycomb-like Network of Li_2_[Ni(cyclam)]_3_[W(CN)_8_]_2_·24H_2_O (**3**)

**Figure 4:**
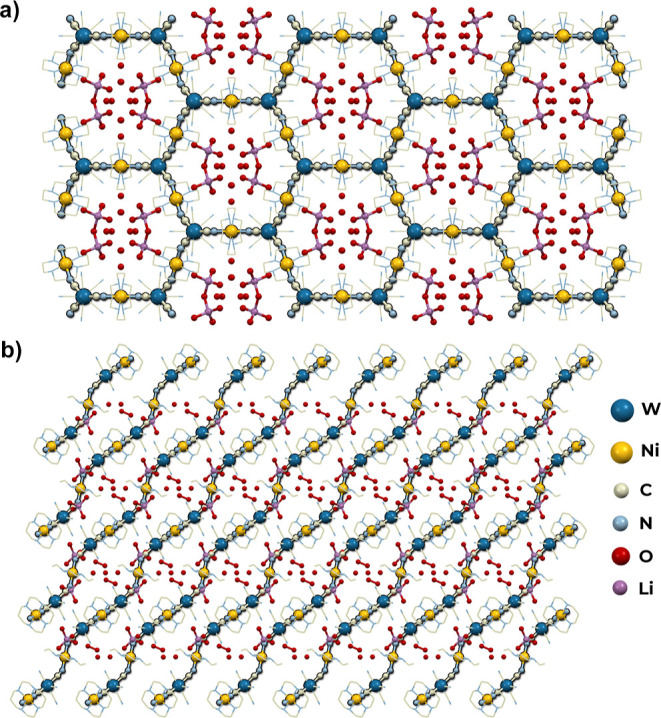
Structure of **3**. View along the channels in the [001] direction (a) and view along the [010] direction showing parallel layers (b).

Li_2_[Ni(cyclam)]_3_[W(CN)_8_]_2_·24H_2_O (**3**) crystallizes in the monoclinic system, the space group *C*2/*m*. The asymmetric unit (Figure S8) is composed of one-half of the [W(CN)_8_]^4–^ ion located in a special position on the mirror plane, one-half of the [Ni(cyclam)]^2+^ complex (Ni2) located at an inversion center, and one-quarter of the nickel complex (Ni1) located at the intersection of mirror plane and two-fold axis. It also contains the lithium ion, disordered over two positions, surrounded by four water molecules, one of which is located on a mirror plane. There are four other water molecules, one located in a general position (O5), two on a mirror plane (O7, O8), and one on a two-fold axis (O6). Each anionic [W(CN)_8_]^4–^ building block is connected by CN bridges to three [Ni(cyclam)]^2+^ cations, acting as a three-connected network node. The coordination geometry of tungsten centers is approximately square antiprismatic (*D*_4d_, CShM = 0.396, Table S1). The nickel cations are equatorially coordinated by N-atoms of cyclam and connected to two tungsten centers through CN bridges, acting as cationic linear linkers of the geometry of slightly disordered octahedron (*O*_h_, CShM = 0.124 and 0.159, Table S2). The cyanido bridges are bent with the angle of Ni–N–C below 160° (Table S4). The structure of **3** is composed of two-dimensional layers of honeycomb-like topology with Schläfli symbol 6^3^ (Figure 4). The distance between parallel slightly corrugated layers located on the (201) planes is 7.65 Å. They are intersected by one-dimensional channels filled with crystallization water molecules and lithium cations that compensate the negative charge of the coordination skeleton. The channels take up 29.8% of the unit cell volume and run along in the *c* crystallographic direction, crossing the coordination layers at an angle of 49°, which is related to the relative relocation of the neighboring layers by 6.65 Å in the [10–2] crystallographic direction. Each lithium cation is surrounded by four water molecules in geometry close to an ideal tetrahedron (*T*_d_, CShM = 0.286, Table S3). The distortion is probably caused by one water molecule (O2) which is shared between two lithium cations resulting in elongation of the Li1–O2 distance. Moreover, there is a net of hydrogen bonds between water molecules, terminal cyanido ligands, and NH-groups of cyclam that additionally stabilize the network. The compound **3** is part of the isotypic family of honeycomb-like networks based on the [M(CN)_8_]^4–^ (M = Nb, Mo) and [Ni(cyclam)]^2+^ complex containing guest ions (NH_4_^+^, Li^+^, Na^+^, Mg^2+^, Ca^2+^, Sr^2+^, Ba^2+^).^29,32,33^

#### 3D Two-fold Interpenetrating Network of [Ni(cyclam)]_5_[Ni(CN)_4_][W(CN)_8_]_2_·11H_2_O (**4**)

**Figure 5:**
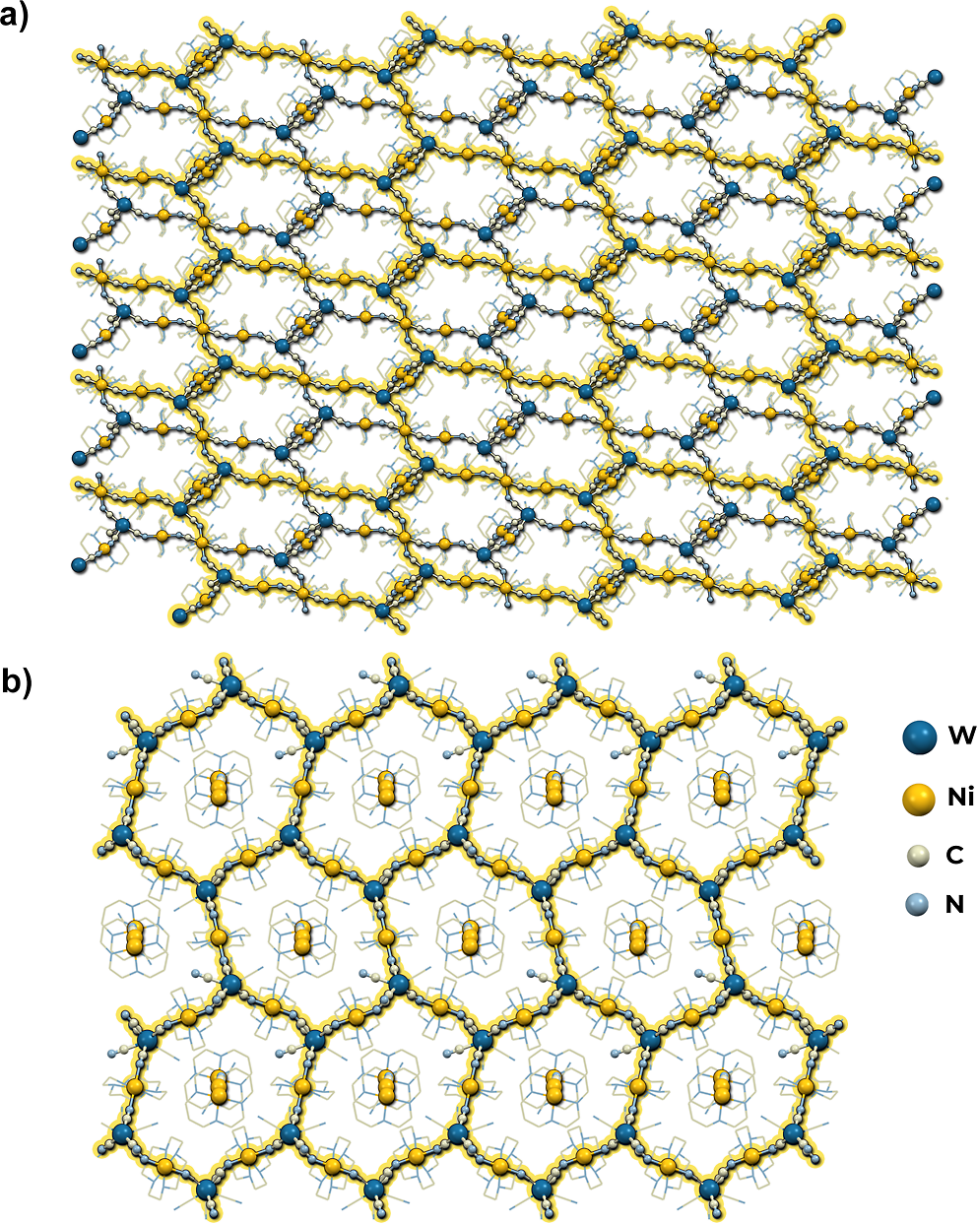
Structure of **4**. View along the [010] direction (a) showing two interpenetrating sub-networks (one marked by yellow highlighting) and view of a single honeycomb-like layer on the (−101) plane with linkers of the second sub-network passing through the hexagonal ring (b). Crystallization water omitted for clarity.

[Ni(cyclam)]_5_[Ni(CN)_4_][W(CN)_8_]_2_·11H_2_O (**4**) crystallizes in the monoclinic system, the space group *P*2_1_/*n*. The coordination network is composed of three building units: cationic [Ni(cyclam)]^2+^, anionic [W(CN)_8_]^4–^, and [Ni(CN)_4_]^2–^. The asymmetric unit presented in Figure S9 consists of the [Ni(cyclam)]^2+^ cation (Ni2) and [Ni(CN)_4_]^2–^ anion (Ni4) both of the occupancy of one-half in special positions on the inversion centers, as well as one [W(CN)_8_]^4–^ anion, two [Ni(cyclam)]^2+^ cations (Ni1, Ni3), and five and a half crystallization water molecules in general positions. Each [W(CN)_8_]^4–^ anion forms four cyanide bridges to the [Ni(cyclam)]^2+^ complexes, acting as a four-connected network node of the geometry close to an ideal triangular dodecahedron (*D*_2d_, CShM = 0.372, Table S1). The cationic [Ni(cyclam)]^2+^ complexes, axially bound by two CN bridges, show slightly disordered octahedral geometry (Table S2). The coordination of [Ni(CN)_4_]^2–^ is close to an ideal square planar geometry (*D*_4h_, CShM = 0.185, Table S3). Both cationic and anionic complexes of nickel ions act as linear linkers. The structure of **4** consists of two separate 3D sub-networks of diamond-like topology with Schläfli symbol 6^6^ (Figure 5). Each sub-network is composed of 2D honeycomb-like layers build of [Ni(cyclam)]^2+^ and [W(CN)_8_]^4–^ building blocks that form hexagonal rings, much more distorted than in the case of compound **3**, which is reflected in the low Ni–N–C angle values (Table S4). Thus, the whole two-fold structure could be described as the set of alternating layers that lay on the ⟨−101⟩ crystallographic planes. The layers of one sub-network are connected through long trimetallic linkers composed of {[Ni(cyclam)]^2+^–[Ni(CN)_4_]^2–^–[Ni(cyclam)]^2+^} units. Those bridging units pass through the centers of hexagonal openings of the other sub-network. The distance between the honeycomb-like layers of one sub-network is equal to 25.01 Å. There are isolated cavities in the structure filled with crystallization water molecules that take up 10.1% of the unit cell volume. The water molecules create a net of hydrogen bonds connected with terminal CN ligands of polycyanidometallates. The compound **4** is isostructural with an analogous network based on the [Mo(CN)_8_]^4–^ building block.^33^

#### 1D Straight Chain of [Ni(cyclam)][Ni(CN)_4_]·2H_2_O (**5**)

**Figure 6:**
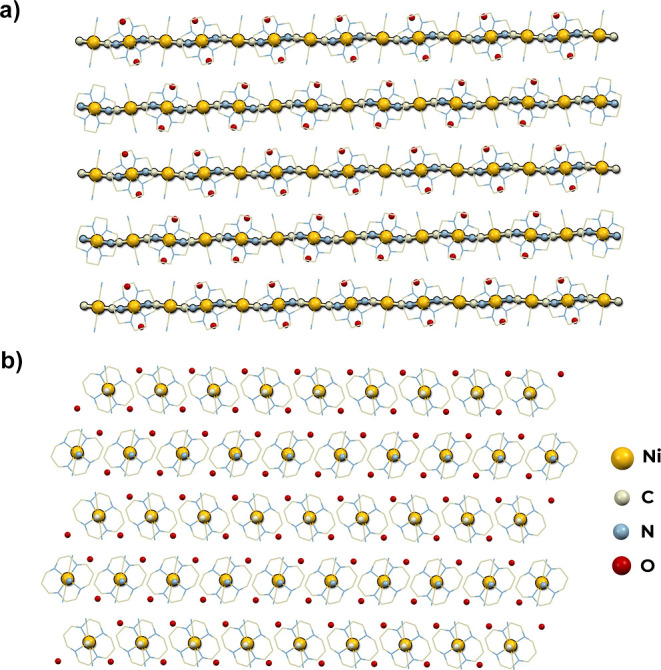
Structure of **5**. View along the [100] direction (a) and along the coordination chains in the [−110] direction (b).

[Ni(cyclam)][Ni(CN)_4_]·2H_2_O (**5**) crystallizes in the triclinic system, the space group *P*1̅. The structure is composed of cationic [Ni(cyclam)]^2+^ and anionic [Ni(CN)_4_]^2–^ building blocks arranged in alternating chains. All metal ions occupy special positions at the centers of inversion. There are two independent positions of the [Ni(cyclam)]^2+^ complex (Ni2, Ni4) and two independent positions of the [Ni(CN)_4_]^2–^ complex (Ni1, Ni3) (Figure S10). The structure also contains two water molecules in general positions. The cationic [Ni(cyclam)]^2+^ complexes, axially bound by N-atoms of cyanido ligands show the smallest distortion of octahedral geometry among structures **1–5** (*O*_h_, CShM = 0.114 (Ni2) and 0.109 (Ni4), Table S2). The [Ni(CN)_4_]^2–^ ions are characterized by almost an ideal square planar geometry (*D*_4h_, CShM = 0.003 (Ni1) and 0.002 (Ni3), Table S3). The structure of **5** consists of one-dimensional chains of alternating cyanido-bridged [Ni(cyclam)]^2+^ and [Ni(CN)_4_]^2–^ complex ions that runs along the [−110] direction (Figure 6). The coordination chains are slightly undulating with the angle of Ni–N–C of 165.6°. The distance between chains in the *c* crystallographic direction is equal to 7.84 Å. The crystallization water molecules form hydrogen bonds only with N-atoms of terminal cyanido ligands and NH-groups of cyclam, resulting in the formation of a three-dimensional supramolecular network. In contrast to **5**, the earlier characterized related chains [Ag(cyclam)][Pt(CN)_4_] and [Cu(cyclam)][M(CN)_4_] (M = Ni, Pd, Pt) do not contain crystallization water and their coordination skeleton has a zigzag shape.^40,41^

### Thermal Stability and Sorption Properties

As described above, the crystalline samples of **1** and **2** collapse and decompose upon even short contact with pure water. Therefore, after the crystals are filtered from the mother liquor, their surface is contaminated with lithium chloride, which is extremely hygroscopic and creates various hydrates depending on the relative humidity.^42^ TGA for both compounds (Figure S11) shows sharp mass loss beginning under dry argon flow at ambient temperature. This initial stage is composed of two unresolved steps with T_max_ at 44 and 79 °C (**1**) or 47 and 63 °C (**2**). From 95 °C (**1**) or 65 °C (**2**) to about 150 °C, a stability phase is observed. Then, a further well-defined mass loss step with *T*_max_ of 160 °C is observed, which is very small for **1** and much larger for **2**. The mass loss below 200 °C can be attributed to dehydration of both the coordination polymer and LiCl. Because of the fact that there is no stability period at the beginning of the experiment and the presence of LiCl impurity, the quantitative interpretation of the TG results is not possible. Larger overall mass loss upon dehydration observed for **1** is consistent with higher amount of crystallization water in this compound. The mass loss step at 160 °C can be attributed mainly to the dehydration of LiCl.^42^ After a long plateau, decomposition of both compounds begins at 250 °C.

Similar to **1** and **2**, the TG curve of **3** (Figure S12) shows sharp two-step initial decrease with *T*_max_ at 39 and 78 °C, indicating that the dehydration begins under the dry argon flow at ambient temperature. After a plateau between 85 and 120 °C, an additional prolonged dehydration step with *T*_max_ of 155 °C occurs, which we believe to be connected with the strong bonding of H_2_O coordinated to the Li^+^ cations. Then, after a short stability period, decomposition begins around 225 °C. For compound **4**, the beginning of dehydration is also not visible in TGA (Figure S12). It is completed in two unresolved steps with *T*_max_ at 60 and 84 °C. From 95 to 250 °C, a long stability period of an anhydrous phase is present, followed by decomposition. The fact that there is no additional dehydration step around 150 °C is consistent with lack of Li^+^ guest cations. TGA of the crystalline sample of **5** shows a flat line in the temperature range of 35–300 °C, indicating that the dehydration process in the dry argon flow is very fast. It is consistent with the EA results, which show that partial dehydration of **5** takes place upon drying in air.

**Figure 7:**
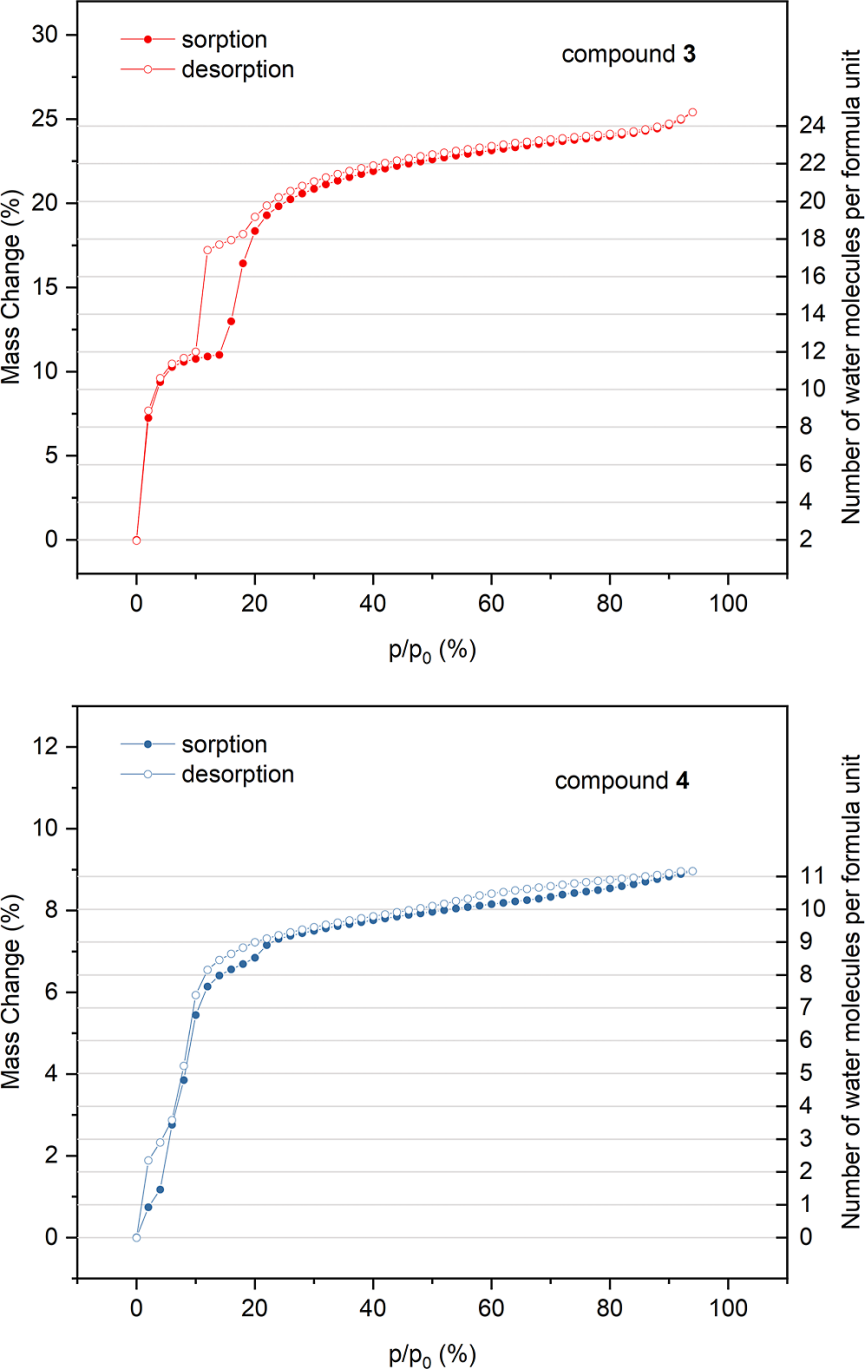
Water sorption isotherms at the temperature of 25 °C for compound **3** (top) and **4** (bottom). Scale on the right indicates the approximate number of crystallization water molecules per formula unit.

The water sorption properties of samples **3** and **4** were studied using the dynamic vapor sorption gravimetric method at a constant temperature of 25 °C and variable relative humidity (RH). The isotherms showing hydration and dehydration processes are presented in Figure 7. Each measurement step in the relative humidity range of 0–94% was performed until the stable sample mass was achieved. The amount of crystallization water molecules in fully hydrated phases designated on the basis of measured isotherms corresponds to the structural models of **3** and **4**. The desorption isotherm of **3** shows three distinct steps, indicating the existence of metastable phases containing 18 H_2_O at 12–16% RH and 12 H_2_O at 6–10% RH. In the dehydrated under dry nitrogen flow phase, there are still two water molecules per formula unit, which are probably strongly bound to lithium cations and cannot be removed at ambient temperature. Taking into account that in the original structure of **3**, there are seven water molecules bound to two Li^+^ per formula unit, we assume that upon dehydration structural transformation with the rearrangement of the Li^+^ coordination sphere takes place, possibly including formation of semi-coordination bonds to terminal cyanide ligands of [W(CN)_8_]^4–^. The sorption isotherm of **3** shows immediate high water intake at 2% RH, which is characteristic for microporous materials. In contrast to the three-step desorption isotherm, it is composed of just two steps, with the metastable phase, containing 12 H_2_O, between 8 and 14% RH. Due to the difference between the sorption and desorption processes, a small hysteresis loop appears in the 10–18% RH range. In the higher humidity region, the sorption and desorption isotherms almost overlap. The desorption isotherm of compound **4** shows that all 11 crystallization water molecules are released under dry nitrogen flow, which corresponds to about 9% of mass change, calculated on the basis of anhydrous polymer mass. The mass decrease upon water desorption is at first very slow and down 12% RH only three water molecules are released. Then, the loss of the remaining eight water molecules takes place in two sharp steps below 10% (∼5H_2_O) and 2% RH (∼3H_2_O). The sorption isotherm of **4** is characterized by a relatively small water intake (∼1H_2_O) up to 4% RH, which reflects the presence of only small isolated cavities in the structure, as opposed to the microporous channels of **3**. Between 4 and 12% RH, a sharp sorption step up to 8H_2_O is observed, followed by a slow and almost linear mass increase over the higher humidity range with a small step at 22% RH. The differences between sorption and desorption processes result in an irregular small hysteresis. The presence of sharp steps and hysteresis in the isotherms of both compounds indicates that the structures are not rigid, but undergo humidity-driven structural transformations adapting to the changes in the number of guest water molecules. Interestingly, the water sorption isotherms of **3** and **4**, although similar, are not identical to those of their Mo-based isotypic congeners.^33^

### Magnetic Properties

**Figure 8:**
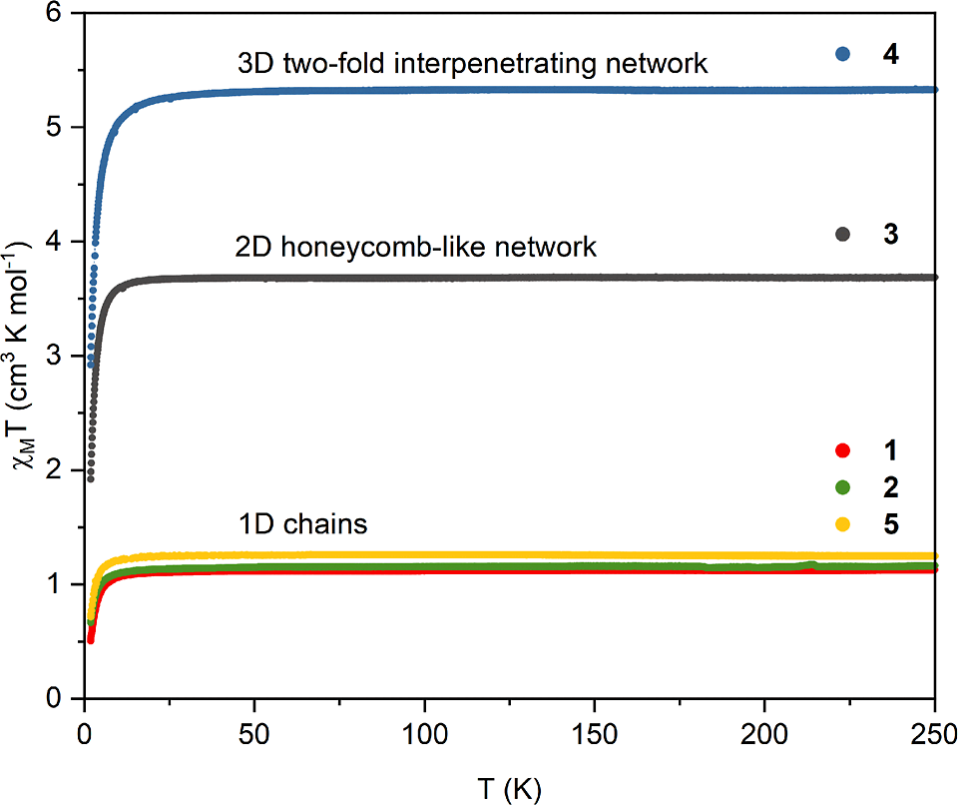
Temperature dependence of χ_M_*T* product for samples **1–5** at a constant magnetic field of 1 kOe.

The magnetic properties of compounds **1–5** were characterized by DC susceptibility measurements at 1 kOe in the temperature range of 1.8–300 K and magnetization measurements at 1.8 K in magnetic fields up to 70 kOe. All microcrystalline samples were carefully protected from dehydration by immersion in pure water or lithium chloride solution of the same concentration as used in their syntheses. Every compound is composed of paramagnetic [Ni(cyclam)]^2+^ complex ions (*S* = 1, *g* = 2.2) that are separated by diamagnetic [W(CN)_8_]^4–^ or [Ni(CN)_4_]^2–^ (in the case of **4** and **5**). Thus, the χ_M_*T* products show a constant value from about 20 K up to 250 K, which is typical for paramagnetic behavior (Figure 8).

The experimental values of high-temperature χ_M_*T* products are in good agreement with the theoretical ones related to the respective chemical formulas and an integer number of Ni^II^ ions with *S* = 1 and *g* = 2.2. The decrease of χ_M_*T* at low temperature is connected with zero field splitting effects and the presence of weak antiferromagnetic interactions between nickel(II) centers through diamagnetic polycyanidometallates. The magnetization curves also show typical paramagnetic run and do not saturate in the field of 70 kOe (Figures S13–S16). Moreover, the magnetic properties of **3** and **4** well corresponds to the characteristics of their octacyanidomolybdate(IV) congeners.^33^

### Photomagnetic Investigations

The light-induced magnetic experiments were performed for a fully hydrated sample of Li_2_[Ni(cyclam)]_3_[W(CN)_8_]_2_·24H_2_O (**3**) and for its octacyanidomolybdate(IV) congener Li_2_[Ni(cyclam)]_3_[Mo(CN)_8_]_2_·24H_2_O (**7**), as well as for the dehydrated phases containing two crystallization water molecules per formula unit (**3**_**deh**_ and **7**_**deh**_). All samples were carefully protected from changing the hydration level.

The sample of **3** shows a negligible change of magnetization even after 26 h of irradiation (Figure S17a), manifested by a negligible lowering of magnetization at high magnetic fields in the *M*(*H*) plot (Figure S17b). The χ_M_*T*(*T*) curves (Figure S17c) before and after irradiation are nearly identical in the 1.8 to 10 K range, after which a negligible artificial decrease for irradiated **3** is observed. Hence, the light-induced effect is not present or negligible in the fully hydrated phase of compound **3**.

**Figure 9:**
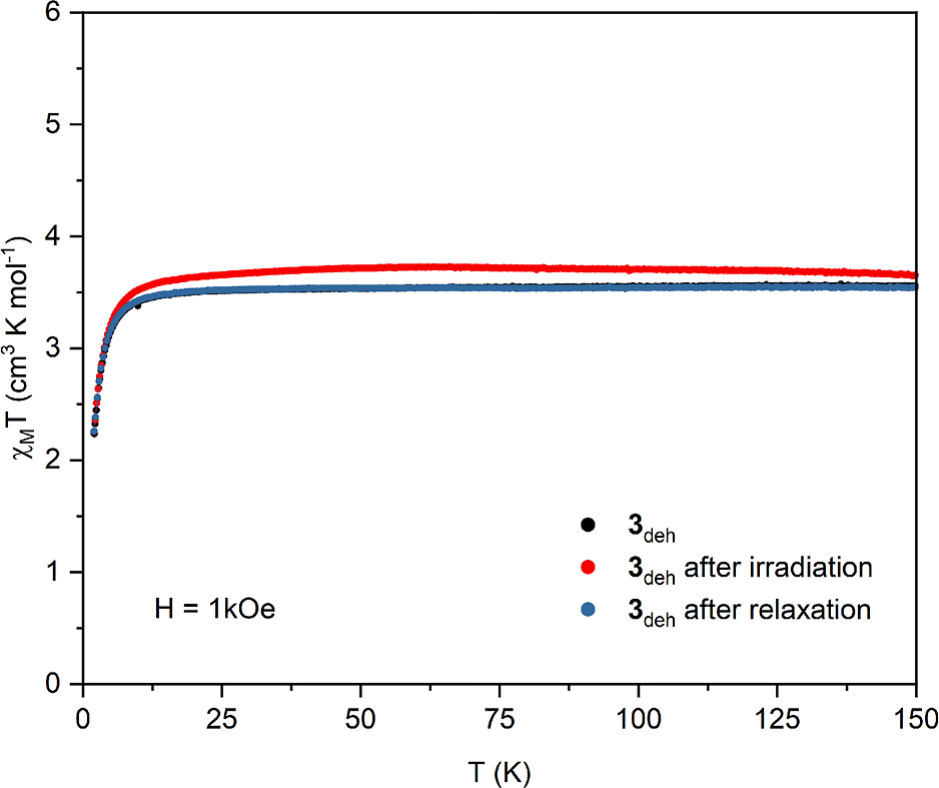
Temperature dependence of χ_M_*T* product in a magnetic field of 1 kOe for dehydrated phase **3**_**deh**_ (black points), after 42 h of blue (450 nm) light irradiation (red points), and after thermal relaxation at 250 K (blue points).

A significant photomagnetic effect can be observed for the dehydrated phase **3**_**deh**_. Figure S18a presents the increase of the magnetic moment during irradiation indicating the conversion of the low-spin diamagnetic [W^IV^(CN)_8_] moiety to the high-spin *S* = 1 species. Such switching was observed before for Mn^II^- and Fe^II^-based systems.^15,16,43^ The *M*(*H*) dependence registered at 1.8 K shows a pronounced lowering of magnetization (ca. 4% at 70 kOe) after 42 h of irradiation (Figure S18b). This suggests that the magnetic interactions between the photo-induced high-spin tungsten(IV) species connected to Ni^II^ ions via CN bridges is most probably antiferromagnetic *J*_WNi_* < 0. The divergence of the *M*(*H*) curves takes place at about 10 kOe. The clear increase of the χ_M_*T* product in the 5–150 K range confirms the persistence of the photo-induced state (Figure 9). The effect caused by the irradiation can be reversed thermally. After heating the sample to 250 K, the initial χ_M_*T*(*T*) and *M*(*H*) curves are fully restored (Figures 9 and S18).

The fully hydrated Li_2_[Ni(cyclam)]_3_[Mo(CN)_8_]_2_·24H_2_O (**7**) shows small magnetization changes in response to 28 h of irradiation (Figure S19a), with slightly higher χ_M_*T* values in the 1.8–50 K range as compared to the “dark” state (Figure S19b). Slightly higher magnetization is also observed in the *M*(*H*) plot recorded after irradiation (Figure S19c). As in the case of **3**_**deh**_, the observed photomagnetic effect is most probably caused by the change of the spin state of the Mo^IV^ centers accompanied by the cyanido ligand dissociation.^13^ After relaxation at 250 K, the *M*(*H*) and χ_M_*T*(*T*) plots overlap perfectly with those recorded before irradiation, hence photo-induced changes are reversible. Additional analysis is hindered by a very small change of the magnetization caused by light irradiation.

**Figure 10:**
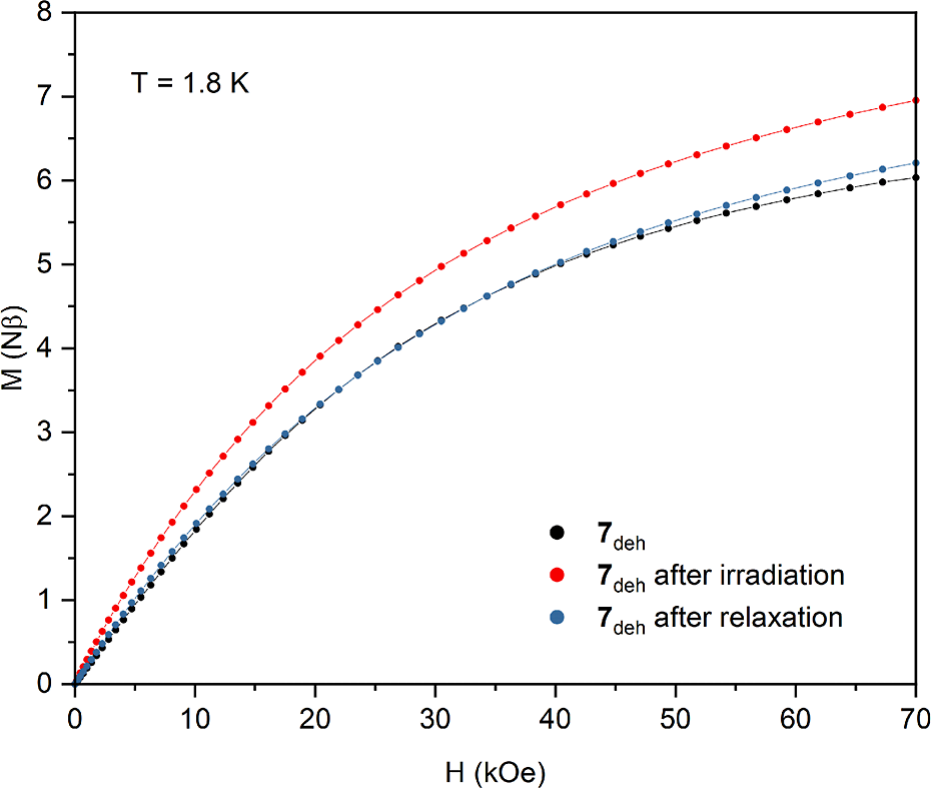
Magnetic field dependence of magnetization at 1.8 K for dehydrated phase **7**_**deh**_ (black points), after 16 h of blue (450 nm) light irradiation (red points), and after thermal relaxation at 250 K (blue points).

**Figure 11:**
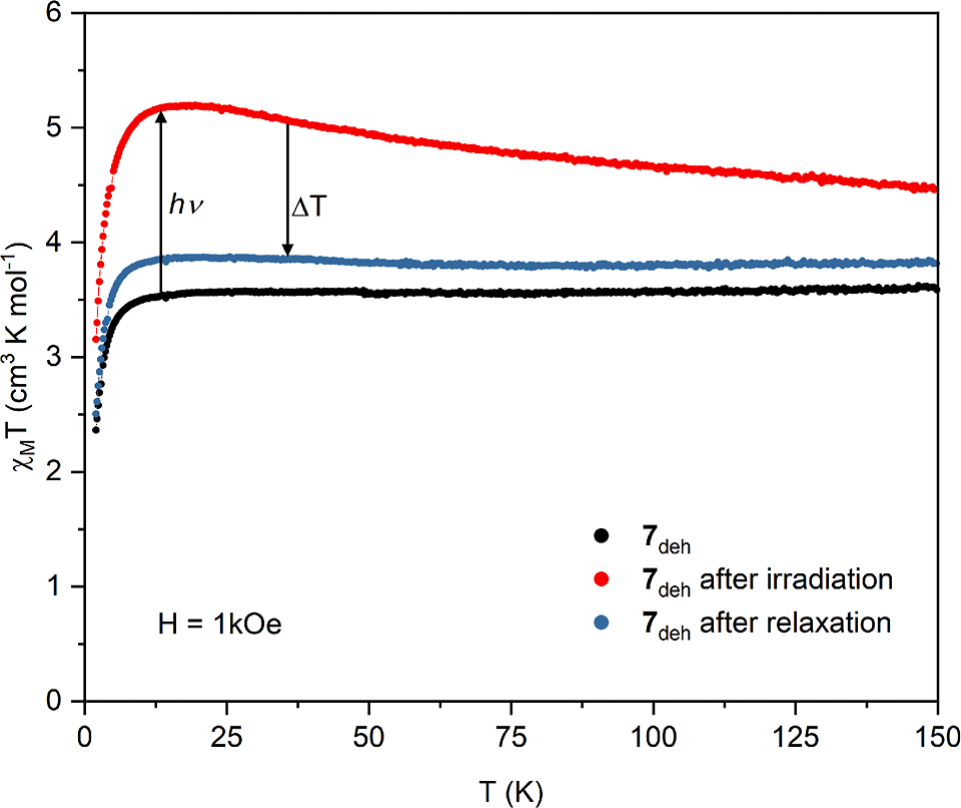
Temperature dependence of χ_M_*T* product in a magnetic field of 1 kOe for dehydrated phase **7**_**deh**_ (black points), after 16 h of blue (450 nm) light irradiation (red points), and after thermal relaxation at 250 K (blue points).

The strongest photomagnetic effect was observed after dehydration of compound **7** resulting in the Li_2_[Ni(cyclam)]_3_[Mo(CN)_8_]_2_·2H_2_O (**7**_**deh**_) phase. The magnetic moment of **7**_**deh**_ increases in response to blue light excitation leading to much higher *M*(*H*) values in the whole investigated magnetic field range. The magnetization at 70 kOe attains the value of 6.95 Nβ which is about 15% larger than that before the irradiation (Figures 10 and S20). The χ_M_*T*(*T*) curve for the irradiated sample shows significant increase in comparison to the “dark” one. This can be ascribed to the behavior similar to the light-induced spin state trapping effect in Fe^II^ complexes.^44^ The diamagnetic *S* = 0 Mo^IV^ center undergoes light-induced spin state switching from the low-spin state to the *S* = 1 high-spin state. Such behavior was observed for a Zn_2_Mo complex.^12^ Analysis of both dependences *M*(*H*) and χ_M_*T*(*T*) suggests that the magnetic interactions between the *S* = 1 Ni^II^ and the photo-induced *S* = 1 Mo^IV^ are ferromagnetic *J*_MoNi_* > 0. This is clearly visible in the photo-induced χ_M_*T*(*T*) curve (red points in Figure 11) as a flat maximum at around 15 K. Heating the sample after the photomagnetic experiment to 250 K does not restore the pristine state completely, suggesting a slight decomposition of the compound which was also observed for some octacyanidomolybdate(IV)–manganese(II) photomagnetic systems, where the photodissociation of the CN ligand was behind the photomagnetic switching mechanism (Figure 11).^45^

## Conclusions

Our work is an unprecedented example of crystal engineering of CN-bridged assemblies. We present a record number of six coordination networks of different dimensionality and topology obtained from the same pair of building blocks: [Ni(cyclam)]^2+^ and [W(CN)_8_]^4–^. The products include coordination polymers with negatively charged frameworks compensated by the presence of the Li^+^ cations: 1D Ni–W chains of straight (**1**) and zigzag (**2**) shape and a 2D honeycomb-like network (**3**). Neutral frameworks formed as a result of partial decomposition of the building blocks: a remarkable 3D two-fold interpenetrating network (**4**) and a 1D Ni–Ni chain (**5**) as well as a 3D Ni–W diamond-like network (**6**) characterized before.^27^ The control of topology and product purity in this complicated multi-pathway reaction system is realized by addition of LiCl electrolyte and temperature adjustment. Moreover, for the first time we present the photomagnetic switching of octacyanidotungstate(IV) and octacyanidomolybdate(IV) photomagnetic chromophores embedded into the nickel(II)-based coordination polymers (**3**, **7**). All previous examples involve other combinations of metal ions: Cu^II^–M^IV^(CN)_8_,^46,47^ Zn^II^–M^IV^(CN)_8_,^12,48^ Mn^II^–M^IV^(CN)_8_,^14,49^ Fe^II^–M^IV^(CN)_8_,^15^ Cd^II^–M^IV^(CN)_8_,^50^ or Pt^IV^–M^IV^(CN)_8_^20^ (M = Mo, W). Similar to Mn^II^–M^IV^(CN)_8_ coordination polymers,^49^ the photomagnetic response of M^IV^(CN)_8_ moieties in the reported Ni^II^–M^IV^(CN)_8_ frameworks is significantly stronger for the dehydrated phases as compared to the pristine ones. This supports the hypothesis that the light-induced spin state switching in M^IV^(CN)_8_ photomagnetic chromophores might be associated with the cyanido ligand photodissociation and the change of the coordination number of the metal center. Additionally, the magnetic interaction of Ni^II^ with the photoexcited M^IV^ through the CN bridges can be either ferromagnetic or antiferromagnetic depending on the bridge geometry. Similar observations were made for Ni^II^–M^V^(CN)_8_ (M = Mo or W)^26,51−55^ and Ni^II^–Nb^IV^(CN)_8_ magnetic coordination frameworks,^27,29,32,56^ where the second metal center M^V^ or Nb^IV^ is an *S* = 1/2 ion.

## Supporting Information Available

Detailed crystallographic data, additional structure diagrams, PXRD data, thermogravimetric characteristics, and magnetic data (PDF)
