# Supplementary material for: Crystal Engineering and Photomagnetic Studies of CN-Bridged Coordination Polymers Based on Octacyanidometallates(IV) and [Ni(cyclam)]2+
Source: Inorg Chem. 2022 Aug 23;61(35):13817–28. doi: 10.1021/acs.inorgchem.2c01629 (PMC9455600; doi:10.1021/acs.inorgchem.2c01629)
Supplement: Supplementary file 1 — ic2c01629_si_001.pdf [file ic2c01629_si_001.pdf]

Supporting Information for

Crystal engineering and photomagnetic studies of CN-bridged coordination polymers based on octacyanidometallates(IV) and $[Ni(cyclam)]^{2+}$

Michał Heczko, Ewa Sumińska, Dawid Pinkowicz, Beata Nowicka*

Faculty of Chemistry, Jagiellonian University, Gronostajowa 2, 30-387 Kraków, Poland

*beata.nowicka@uj.edu.pl

Contents:

Figure S1. Powder X-ray diffraction patterns of **1** 2

Figure S2. Powder X-ray diffraction patterns of **2** 2

Figure S3. Powder X-ray diffraction patterns of **3** 3

Figure S4. Powder X-ray diffraction patterns of **4** 3

Figure S5. Powder X-ray diffraction patterns of **5** 4

Table S1. Continuous Shape Measure parameters for the octa-coordinated tungsten centres in compounds **1**-**4** 4

Table S2. Continuous Shape Measure parameters for the hexa-coordinated nickel centres in compounds **1**-**5** 4

Table S3. Continuous Shape Measure parameters for the tetra-coordinated nickel and lithium centres in compounds **1**-**5** 5

Figure S6. Asymmetric unit of **1** 5

Figure S7. Asymmetric unit of **2** 6

Figure S8. Asymmetric unit of **3** 6

Figure S9. Asymmetric unit of **4** 7

Figure S10. Asymmetric unit of **5** 7

Table S4. Selected interatomic distances and bond angles in the structures of **1**-**5**. 8

Figure S11. Thermogravimetric analysis for compound **1** and **2** 9

Figure S12. Thermogravimetric analysis for compound **3** and **4** 9

Figure S13. Magnetic characteristic of **1** 10

Figure S14. Magnetic characteristic of **2** 10

Figure S15. Magnetic characteristic of **4** 11

Figure S16. Magnetic characteristic of **5** 11

Figure S17. Photomagnetic characteristic of **3**. 12

Figure S18. Photomagnetic characteristic of dehydrated compound **3** 13

Figure S19. Photomagnetic characteristic of **7**. 14

Figure S20. Photomagnetic characteristic of dehydrated compound **7** 15

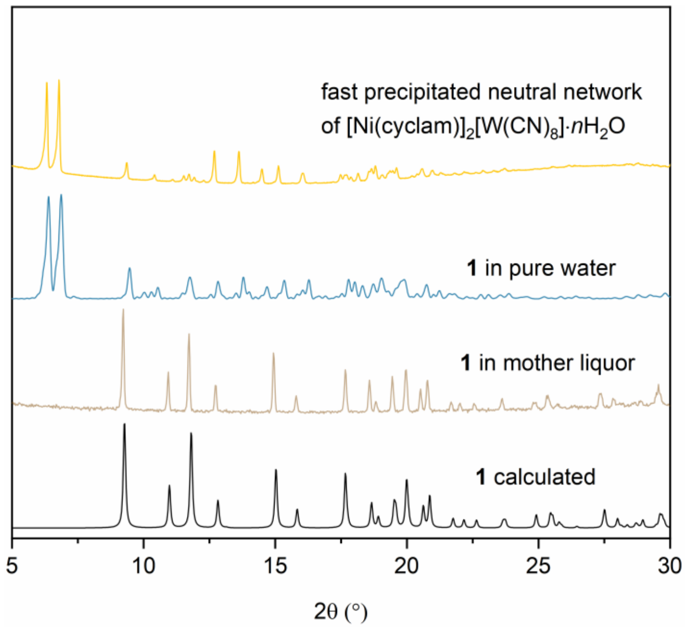

Figure S1. Powder X-ray diffraction patterns of **1**: calculated form SC-XRD model at 120K (bottom), sample in mother liquor, sample immersed in pure water, and pattern of fast precipitated neutral [Ni(cyclam)]$_2$[W(CN)$_8$]·nH$_2$O network in water (top) for comparison.

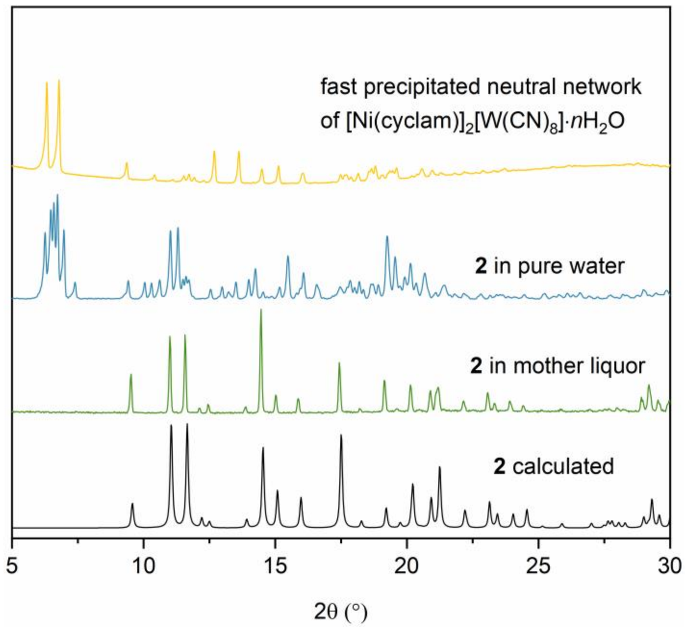

Figure S2. Powder X-ray diffraction patterns of **2**: calculated form SC-XRD model at 120K (bottom), sample in LiCl solution, sample immersed in pure water, and pattern of fast precipitated neutral [Ni(cyclam)]$_2$[W(CN)$_8$]·nH$_2$O network in water (top) for comparison.

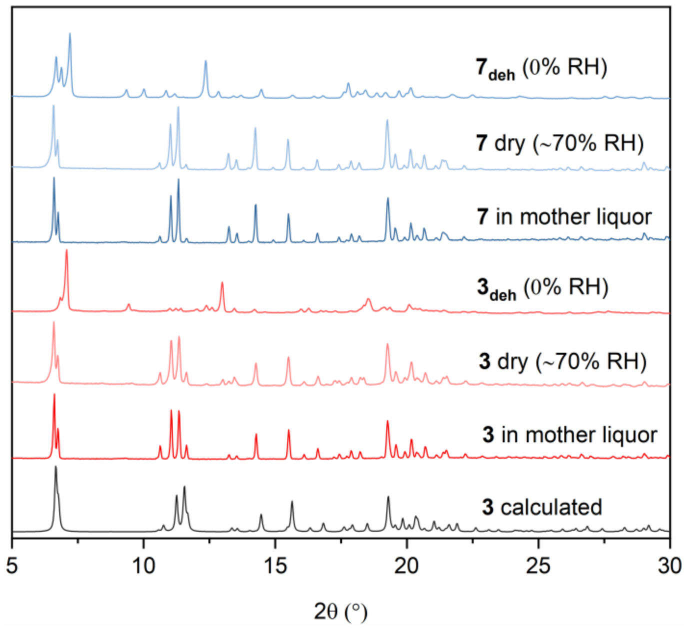

Figure S3. Powder X-ray diffraction patterns of **3**: calculated form SC-XRD model at 100K (bottom), sample in LiCl solution, dry sample stabilized at 70% RH, dehydrated sample at 0% RH, sample of $Li_2[Ni(cyclam)]_3[Mo(CN)_8]_2 \cdot 24H_2O$ (**7**) in LiCl solution, dry sample of **7** at 70% RH, dehydrated **7** at 0% RH (top).

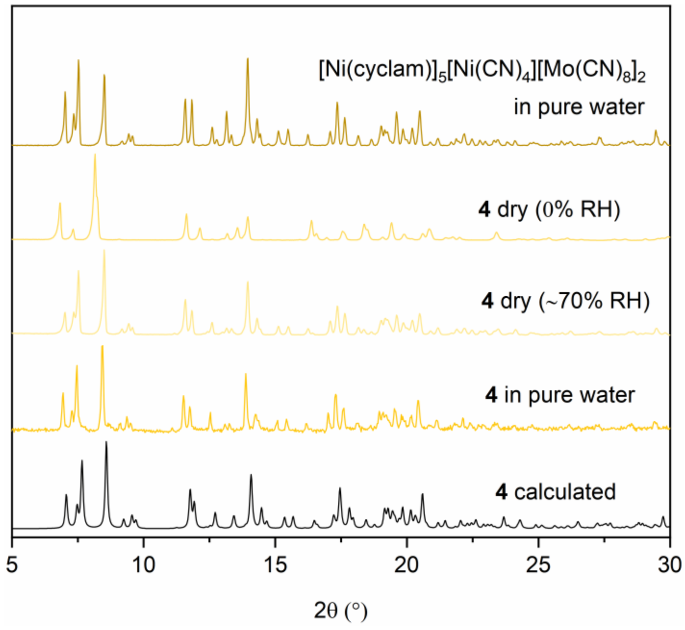

Figure S4. Powder X-ray diffraction patterns of **4**: calculated form SC-XRD model at 100K (bottom), sample in pure water, dry sample at 70% RH, dehydrated sample at 0% RH, and sample of $[Ni(cyclam)]_5[Ni(CN)_4][Mo(CN)_8]_2 \cdot 10H_2O$ in pure water (top) for comparison.

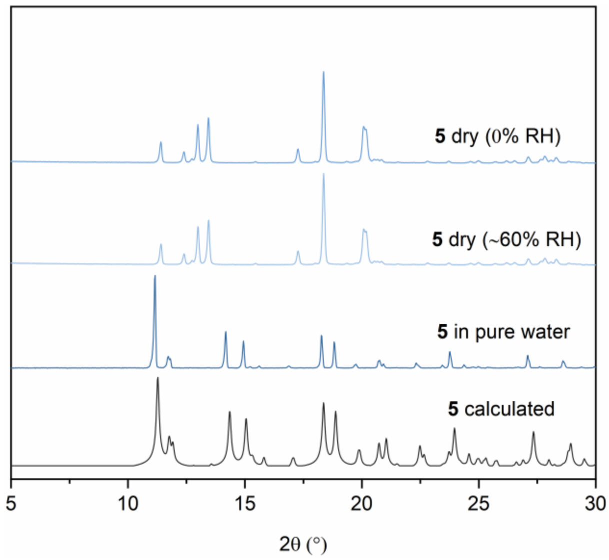

Figure S5. Powder X-ray diffraction patterns of **5**: calculated form SC-XRD model at 100K (bottom), sample immersed in pure water, dry sample stabilized at 60% RH, and dehydrated sample at 0% RH (top).

Table S1. Continuous Shape Measure parameters for the octa-coordinated tungsten centres in compounds **1**-**4**.

		SAPR-8 (D_{4d})	TDD-8 (D_{2d})	BTPR-8 (C_{2v})	JSD-8 (D_{2d})
**1**	W1	**0.166**	2.438	2.195	5.067
**2**	W1	1.820	**0.319**	2.021	2.742
**3**	W1	**0.396**	1.900	1.362	4.427
**4**	W1	2.713	**0.372**	1.983	2.631

CShM = 0 indicates an ideal geometry; SAPR-8 = square antiprism, TDD-8 = triangular dodecahedron, BTPR-8 = biaugmented trigonal prism, JSD-8 = snub disphenoid.

Table S2. Continuous Shape Measure parameters for the hexa-coordinated nickel centres in compounds **1**-**5**.

	**1**	**2**	**3**	**4**	**5**
OC-6 (O_h)	Ni1: 0.172	Ni1: 0.195	Ni1: 0.124<br>Ni2: 0.159	Ni1: 0.169<br>Ni2: 0.114<br>Ni3: 0.206	Ni2: 0.114<br>Ni4: 0.109

CShM = 0 indicates an ideal geometry; OC-6 = octahedron.

Table S3. Continuous Shape Measure parameters for the tetra-coordinated nickel and lithium centres in compounds **1**-**5**.

		SP-4 (D_{4h})	T-4 (T_d)	SS-4 (C_{2v})	vTBPY-4 (C_{3v})
**1**	Li1	26.520	**0.780**	6.601	3.115
**2**	Li1	31.840	**1.955**	6.968	3.478
**3**	Li1	30.540	**0.286**	7.930	3.280
**4**	Ni4	**0.185**	33.457	19.025	34.914
**5**	Ni1	**0.003**	33.335	19.007	34.856
	Ni3	**0.002**	33.335	18.958	34.832

CShM = 0 indicates an ideal geometry; SP-4 = square, T-4 = tetrahedron, SS-4 = seesaw, vTBPY-4 = vacant trigonal bipyramid.

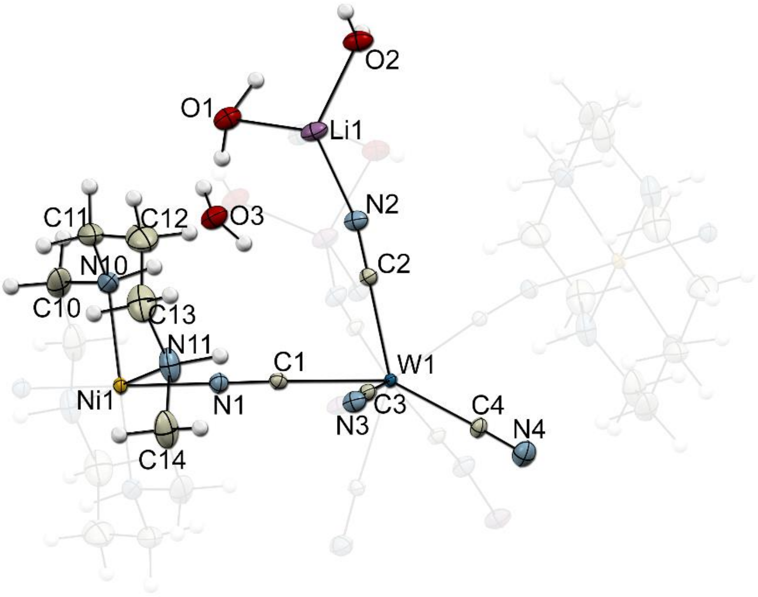

Figure S6. Asymmetric unit of **1**; thermal ellipsoids shown at 50% probability.

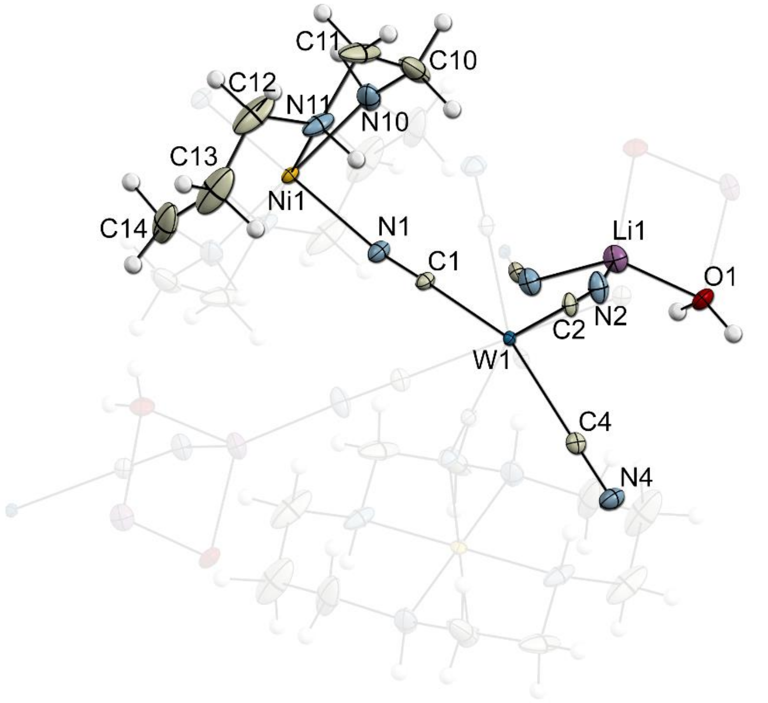

Figure S7. Asymmetric unit of **2**; thermal ellipsoids shown at 50% probability.

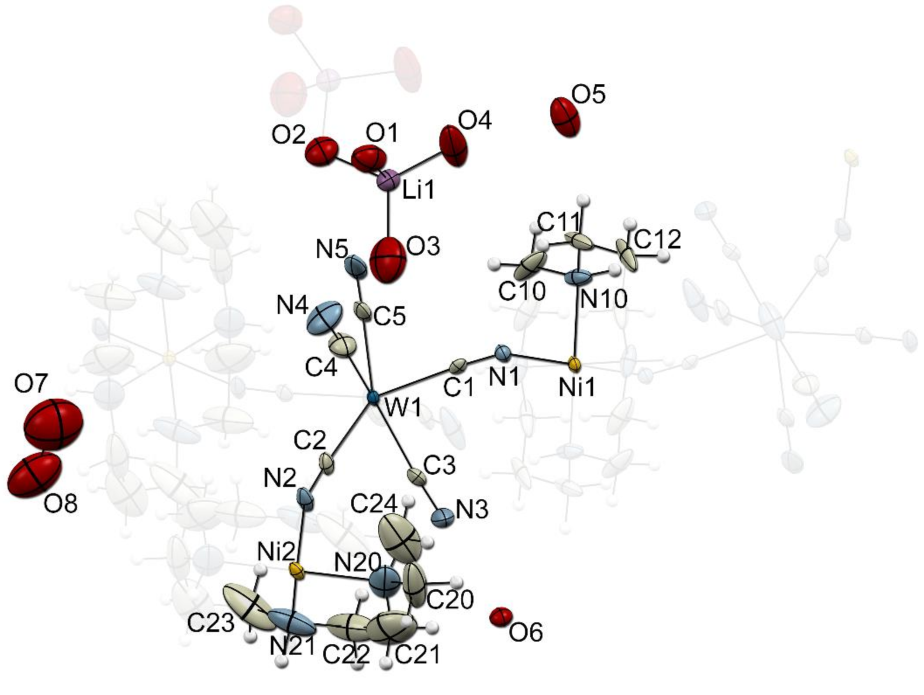

Figure S8. Asymmetric unit of **3**; thermal ellipsoids shown at 50% probability.

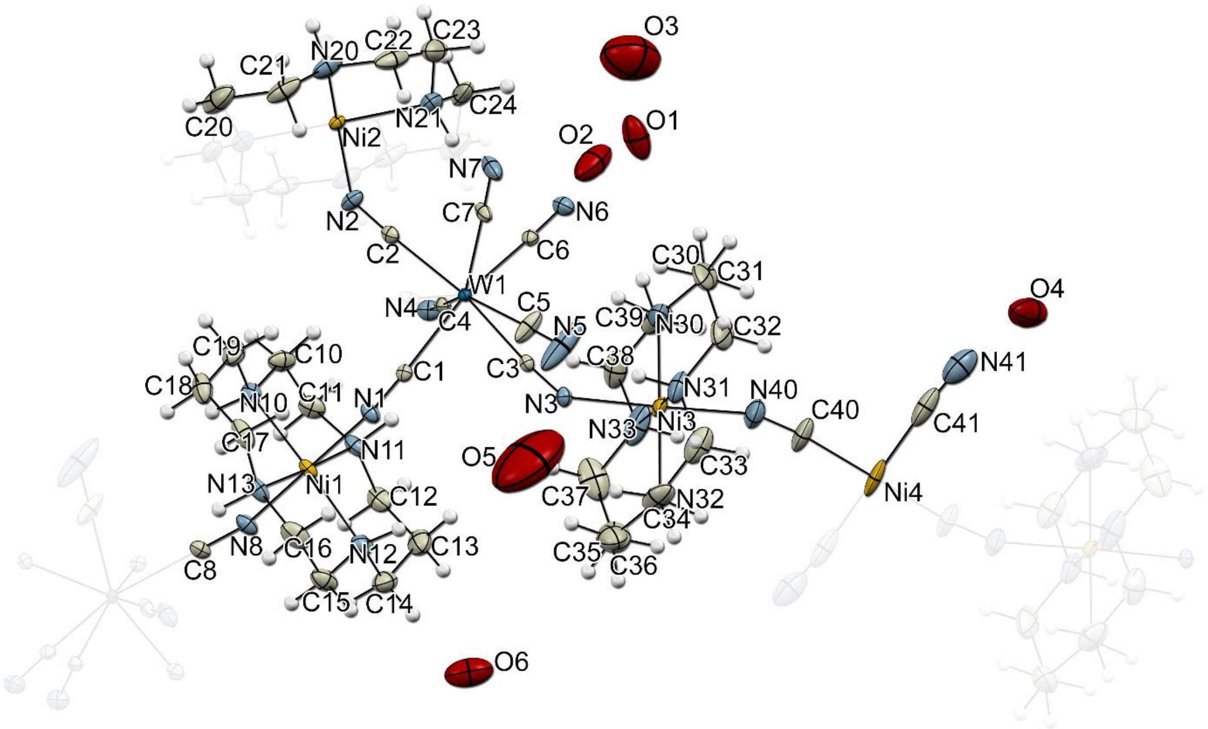

Figure S9. Asymmetric unit of **4**; thermal ellipsoids shown at 50% probability.

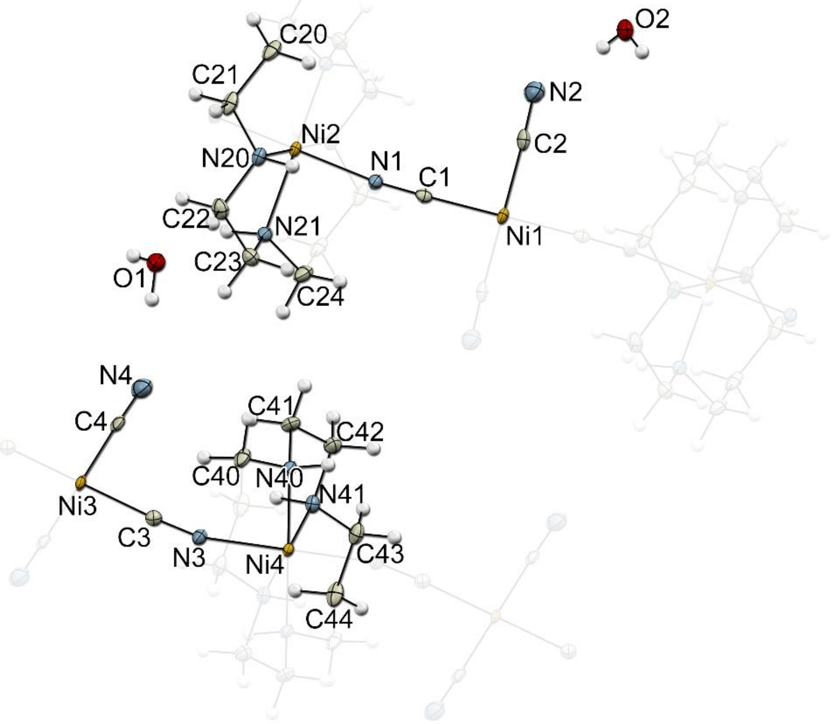

Figure S10. Asymmetric unit of **5**; thermal ellipsoids shown at 50% probability.

Table S4. Selected interatomic distances and bond angles in the structures of **1-5**.

Parameter	**1**	**2**	**3**	**4**	**5**
Distance between metal centers (Å)	Ni1-W1: 5.3258(1)	Ni1-W1: 5.2527(3)	Ni1-W1: 5.1656(4)<br>Ni2-W1: 5.3019(3)	Ni1-W1: 5.2454(5)<br>Ni2-W1: 5.0742(2)<br>Ni3-W1: 5.1447(5)<br>Ni3-Ni4: 5.0031(5)	Ni1-Ni2: 5.0549(4)<br>Ni3-Ni4: 5.0549(4)
Ni-N_{CN} distance (Å)	Ni1-N1: 2.115(2)	Ni1-N1: 2.0689(18)	Ni1-N1: 2.093(8)<br>Ni2-N2: 2.094(6)	Ni1-N1: 2.089(3)<br>Ni2-N2: 2.098(3)<br>Ni3-N3: 2.135(3)<br>Ni3-N40: 2.103(4)	Ni2-N1: 2.096(3)<br>Ni4-N3: 2.099(3)
Ni-N_{cyclam} distance (Å)	Ni1-N10: 2.067(3)<br>Ni1-N11: 2.073(3)	Ni1-N10: 2.092(2)<br>Ni1-N11: 2.068(2)	Ni1-N10: 2.072(7)<br>Ni2-N20: 2.080(10)<br>Ni2-N21: 2.064(10)	Ni1-N10: 2.086(3)<br>Ni1-N11: 2.075(3)<br>Ni1-N12: 2.079(3)<br>Ni1-N13: 2.080(3)<br>Ni2-N20: 2.075(4)<br>Ni2-N21: 2.089(4)<br>Ni3-N30: 2.078(4)<br>Ni3-N31: 2.058(4)<br>Ni3-N32: 2.062(4)<br>Ni3-N33: 2.094(5)	Ni2-N20: 2.080(3)<br>Ni2-N21: 2.076(3)<br>Ni4-N40: 2.086(3)<br>Ni4-N41: 2.072(3)
Ni-N≡C angle (°)	Ni1-N1≡C1: 160.0(2)	Ni1-N1≡C1: 155.47(17)	Ni1-N1≡C1: 149.8(8)<br>Ni2-N2≡C2: 159.8(6)	Ni1-N1≡C1: 157.2(3)<br>Ni1-N8≡C8: 153.7(3)<br>Ni2-N2≡C2: 142.4(3)<br>Ni3-N3≡C3: 144.1(3)<br>Ni3-N40≡C40: 160.8(5)	Ni2-N1≡C1: 165.7(3)<br>Ni4-N3≡C3: 165.6(3)

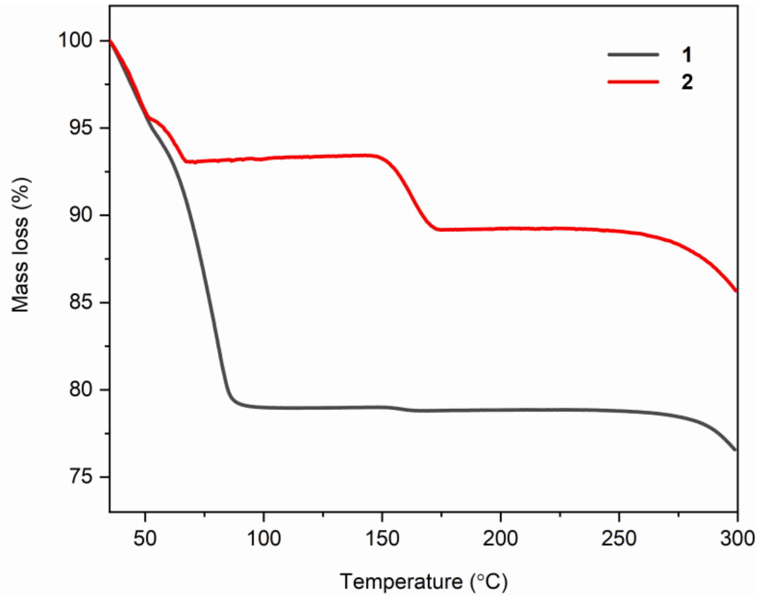

Figure S11. Thermogravimetric analysis for compound **1** and **2** measured under Ar atmosphere with heating rate 2°C/min.

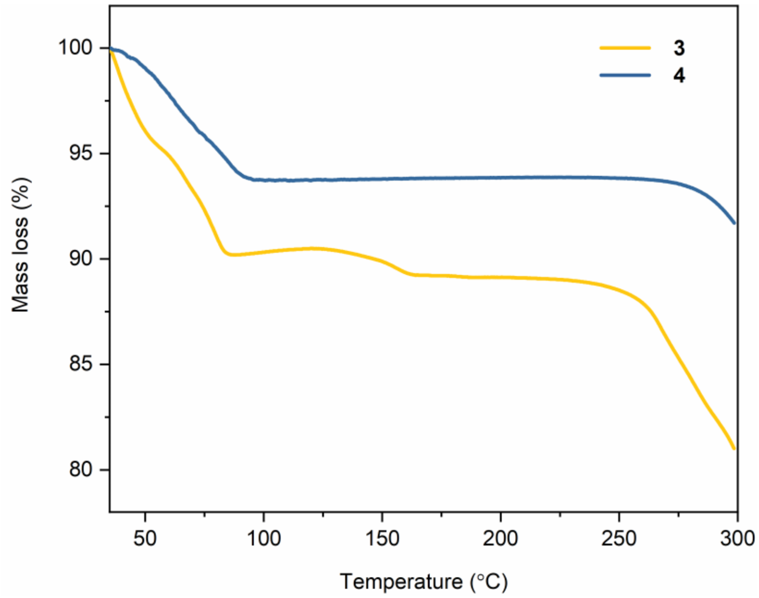

Figure S12. Thermogravimetric analysis for compound **3** and **4** measured under Ar atmosphere with heating rate 2°C/min.

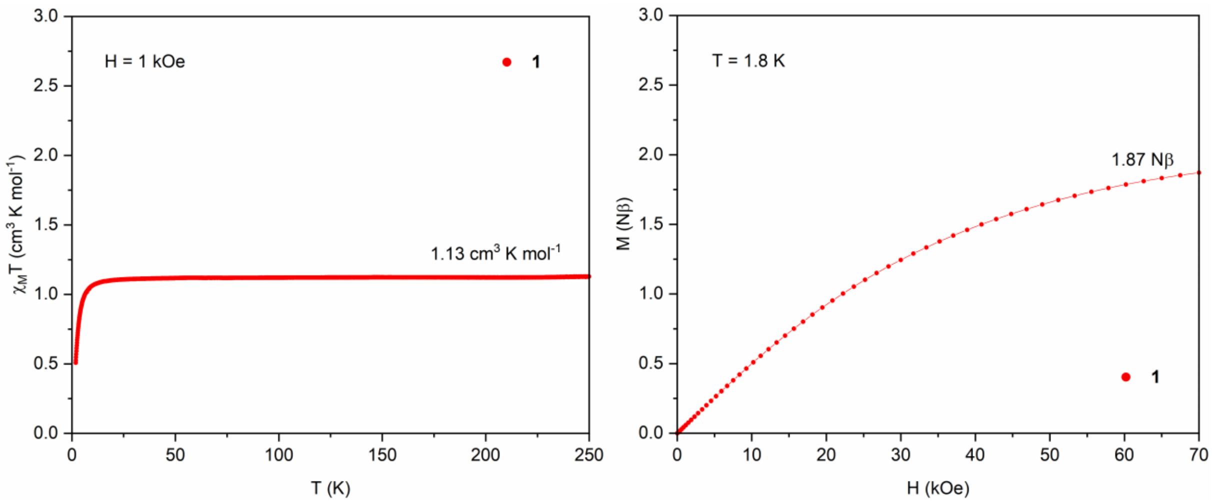

Figure S13. Magnetic characteristic of **1**. χT vs. temperature plot at constant magnetic field of 1 kOe and magnetization vs. magnetic field measurements at 1.8 K. Expected value of high temperature χT product is 1.21 cm^3 K mol^{-1} (Ni(II), s = 1, g = 2.2); M_{sat} = 2.2 Nβ.

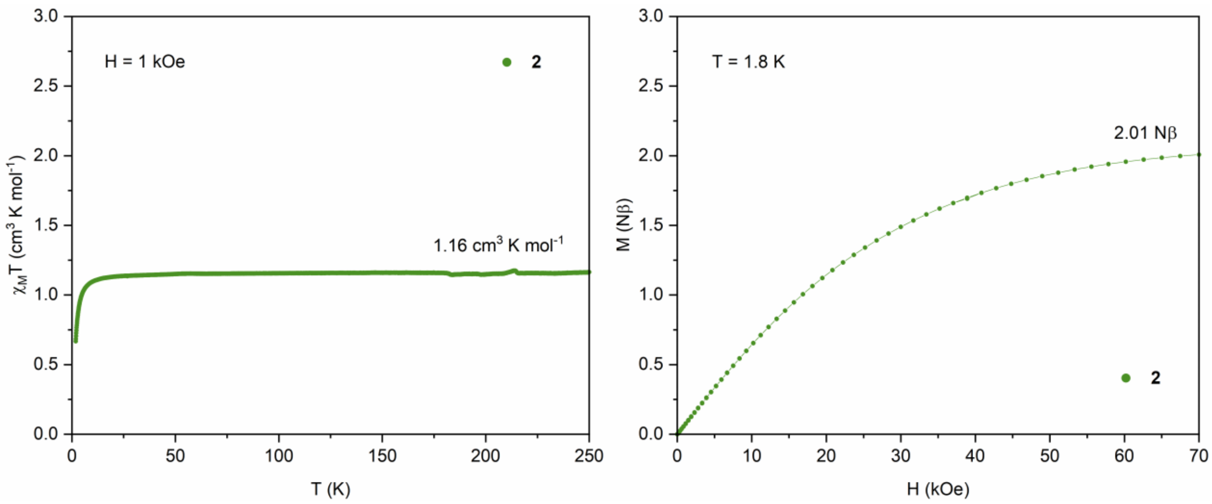

Figure S14. Magnetic characteristic of **2**. χT vs. temperature plot at constant magnetic field of 1 kOe and magnetization vs. magnetic field measurements at 1.8 K. Expected value of high temperature χT product is 1.21 cm^3 K mol^{-1} (Ni(II), s = 1, g = 2.2); M_{sat} = 2.2 Nβ.

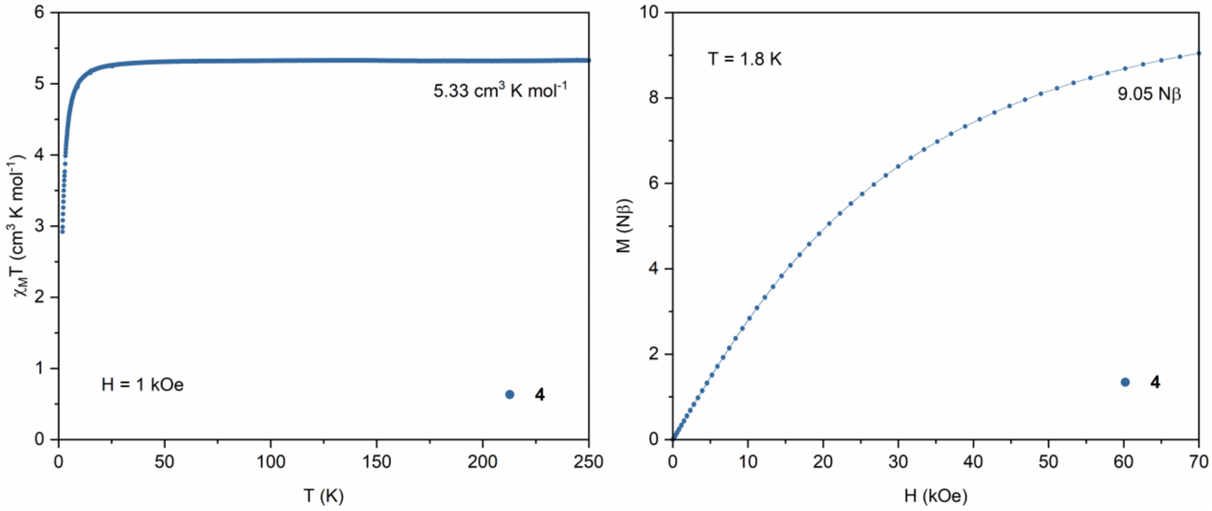

Figure S15. Magnetic characteristic of **4**. χT vs. temperature plot at constant magnetic field of 1 kOe and magnetization vs. magnetic field measurements at 1.8 K. Expected value of high temperature χT product is 6.05 cm^3 K mol^{-1} (Ni(II), s = 1, g = 2.2); M_{sat} = 11 Nβ.

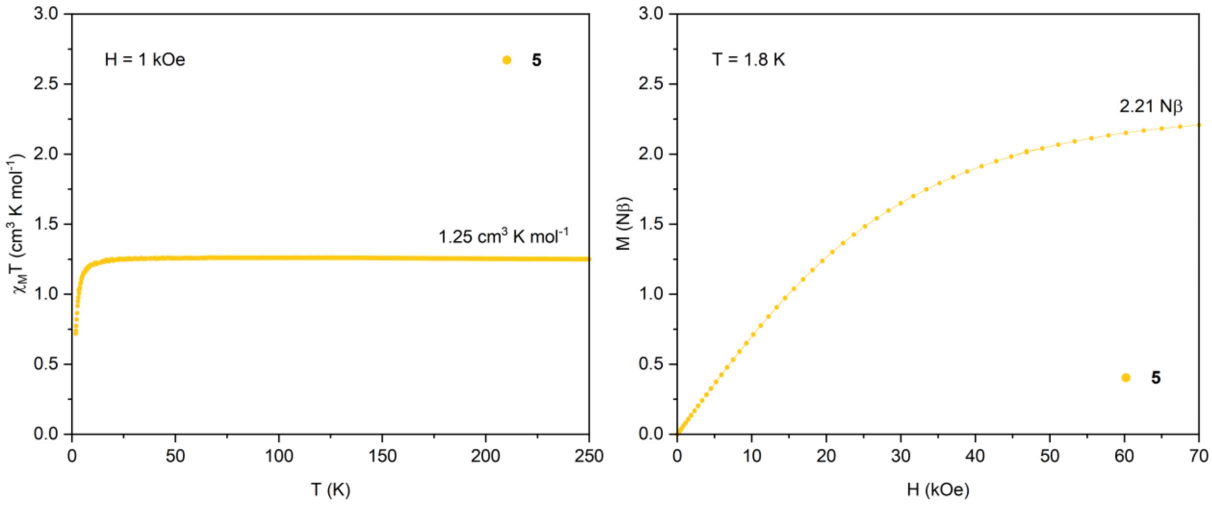

Figure S16. Magnetic characteristic of **5**. $\chi_M T$ vs. temperature plot at constant magnetic field of 1 kOe and magnetization vs. magnetic field measurements at 1.8 K. Expected value of high temperature χT product is 1.21 cm^3 K mol^{-1} (Ni(II), s = 1, g = 2.2); M_{sat} = 2.2 Nβ.

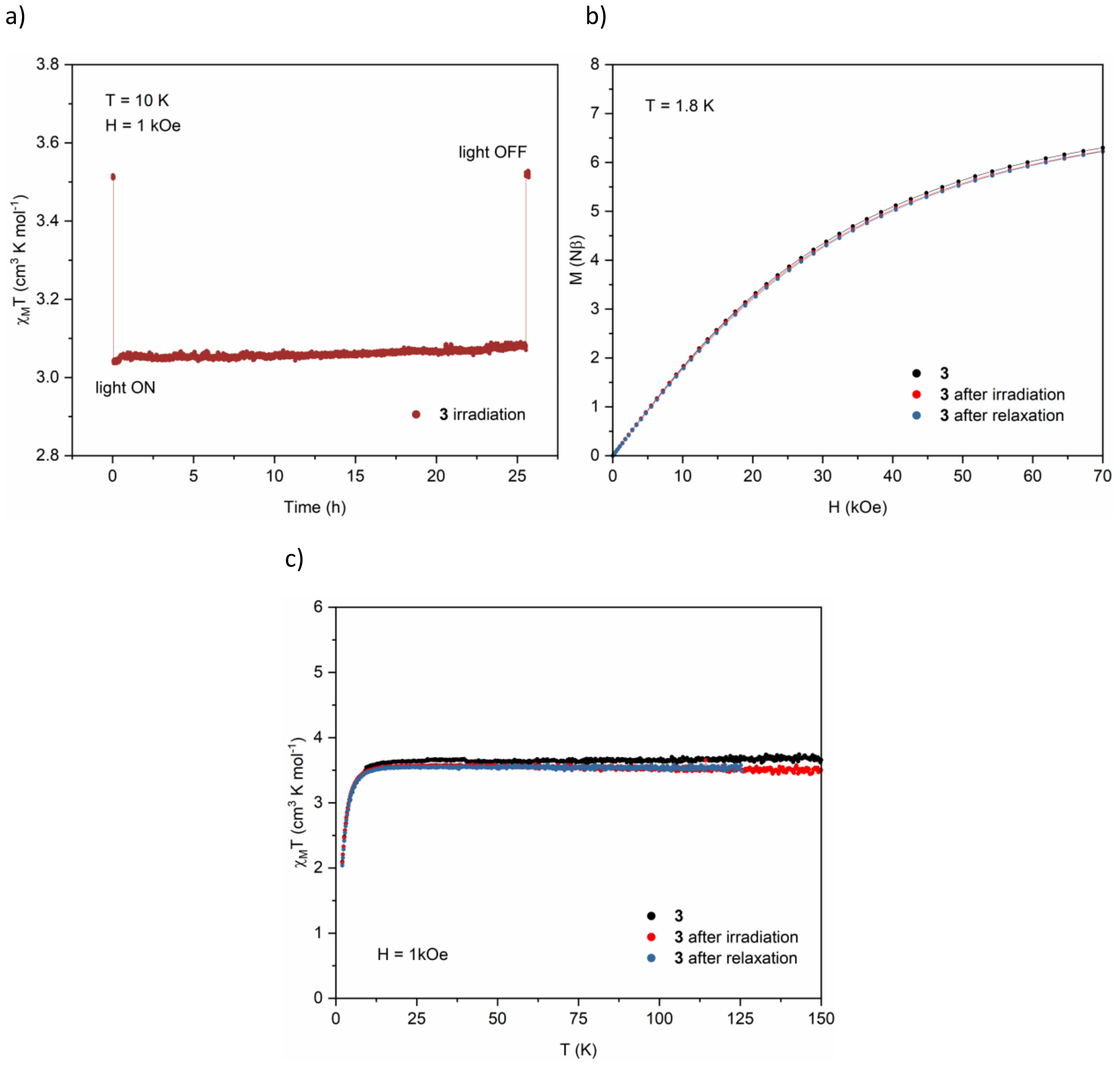

Figure S17. Photomagnetic characteristic of **3**. The irradiation at 10 K under magnetic field of 1 kOe with 405 nm light (a), magnetic field dependence of the magnetization at 1.8 K (b), and temperature dependence of $\chi_M T$ product in magnetic field of 1 kOe (c); bulk sample of **3** indicated as black points, sample after about 26 hours of violet light irradiation (red points), and after thermal relaxation at 250 K (blue points).

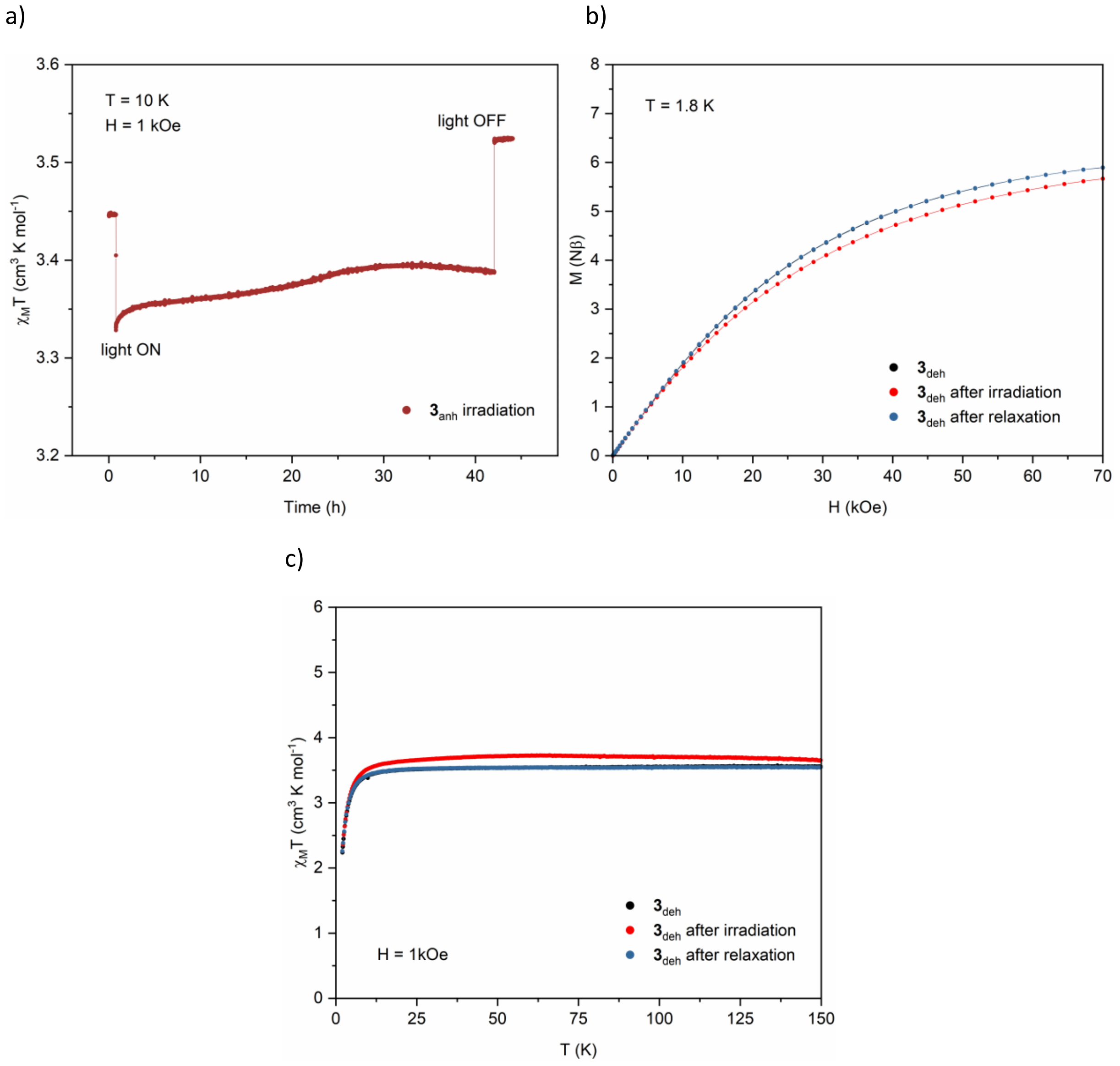

Figure S18. Photomagnetic characteristic of dehydrated compound **3**. The irradiation at 10 K under magnetic field of 1 kOe with 450 nm light (a), magnetic field dependence of the magnetization at 1.8 K (b), and temperature dependence of $\chi_M T$ product in magnetic field of 1 kOe (c); bulk sample of $\mathbf{3_{deh}}$ indicated as black points, sample after about 42 hours of blue light irradiation (red points), and after thermal relaxation at 250 K (blue points).

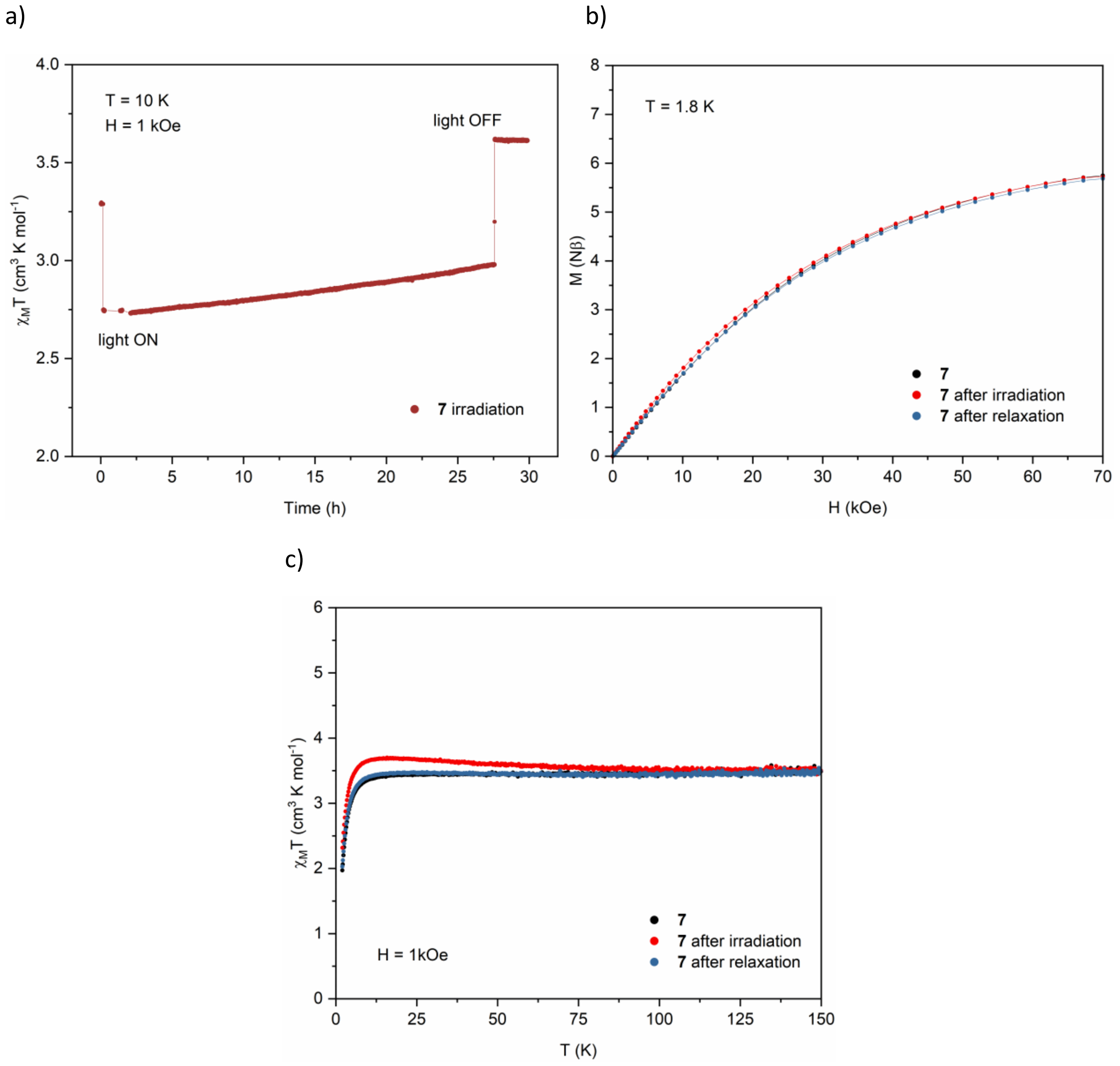

Figure S19. Photomagnetic characteristic of **7**. The irradiation at 10 K under magnetic field of 1 kOe with 405 nm light (a), magnetic field dependence of the magnetization at 1.8 K (b), and temperature dependence of $\chi_M T$ product in magnetic field of 1 kOe (c); bulk sample of **7** indicated as black points, sample after about 28 hours of violet light irradiation (red points), and after thermal relaxation at 250 K (blue points).

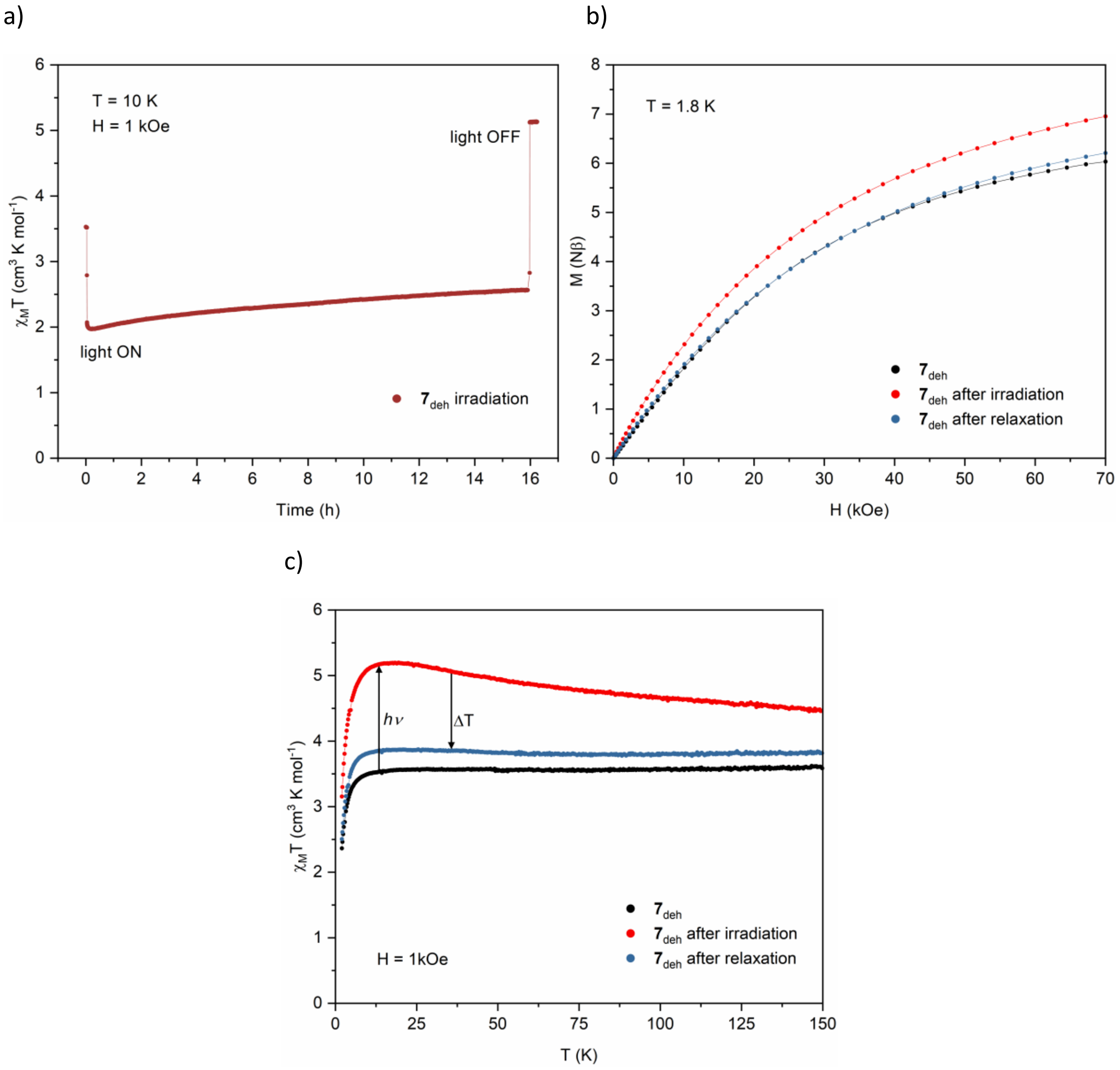

Figure S20. Photomagnetic characteristic of dehydrated compound **7**. The irradiation at 10 K under magnetic field of 1 kOe with 450 nm light (a), magnetic field dependence of the magnetization at 1.8 K (b), and temperature dependence of $\chi_M T$ product in magnetic field of 1 kOe (c); bulk sample of $\mathbf{7_{deh}}$ indicated as black points, sample after about 16 hours of blue light irradiation (red points), and after thermal relaxation at 250 K (blue points).
